# Dissemination and implementation research in dementia care: a systematic scoping review and evidence map

**DOI:** 10.1186/s12877-017-0528-y

**Published:** 2017-07-14

**Authors:** Ilianna Lourida, Rebecca A Abbott, Morwenna Rogers, Iain A Lang, Ken Stein, Bridie Kent, Jo Thompson Coon

**Affiliations:** 1 NIHR CLAHRC South West Peninsula (PenCLAHRC), University of Exeter Medical School, University of Exeter, South Cloisters, St Luke’s Campus, Exeter, EX1 2LU UK; 2 School of Nursing and Midwifery, Plymouth University, Plymouth, UK

**Keywords:** Dementia care, Implementation, Dissemination, Strategies, Scoping review

## Abstract

**Background:**

The need to better understand implementing evidence-informed dementia care has been recognised in multiple priority-setting partnerships. The aim of this scoping review was to give an overview of the state of the evidence on implementation and dissemination of dementia care, and create a systematic evidence map.

**Methods:**

We sought studies that addressed dissemination and implementation strategies or described barriers and facilitators to implementation across dementia stages and care settings. Twelve databases were searched from inception to October 2015 followed by forward citation and grey literature searches. Quantitative studies with a comparative research design and qualitative studies with recognised methods of data collection were included. Titles, abstracts and full texts were screened independently by two reviewers with discrepancies resolved by a third where necessary. Data extraction was performed by one reviewer and checked by a second. Strategies were mapped according to the ERIC compilation.

**Results:**

Eighty-eight studies were included (30 quantitative, 34 qualitative and 24 mixed-methods studies). Approximately 60% of studies reported implementation strategies to improve practice: training and education of professionals (94%), promotion of stakeholder interrelationships (69%) and evaluative strategies (46%) were common; financial strategies were rare (15%). Nearly 70% of studies reported barriers or facilitators of care practices primarily within residential care settings. Organisational factors, including time constraints and increased workload, were recurrent barriers, whereas leadership and managerial support were often reported to promote implementation. Less is known about implementation activities in primary care and hospital settings, or the views and experiences of people with dementia and their family caregivers.

**Conclusion:**

This scoping review and mapping of the evidence reveals a paucity of robust evidence to inform the successful dissemination and implementation of evidence-based dementia care. Further exploration of the most appropriate methods to evaluate and report initiatives to bring about change and of the effectiveness of implementation strategies is necessary if we are to make changes in practice that improve dementia care.

**Electronic supplementary material:**

The online version of this article (doi:10.1186/s12877-017-0528-y) contains supplementary material, which is available to authorized users.

## Background

Dementia is a multi-causal syndrome characterised by progressive deterioration in cognitive abilities and impairment in the ability to perform everyday activities; it can compromise capacity for independent living and lead to needs for care [1]. More than 35 million people live with dementia worldwide and, given that the disease is primarily associated with increasing age, the number is likely to increase in ageing populations [2]. Dementia is now among the most feared conditions in adults aged over 55 [3] and poses a significant economic burden to individuals and healthcare systems with average annual costs over €160 billion in Europe and $150 billion in the US [4, 5].

Perhaps because of this growing cost, dementia has come increasingly to the attention of policymakers (e.g. Department of Health 2015 [6], US Department of Health and Human Services 2016 [7]) who have highlighted the need for more research on prevention, care, and cure as well as for high quality service provision. Despite this, there remains a persistent gap between evidence provision and implementation: currently provided dementia care often does not reflect what research evidence suggests would improve outcomes. There is intermittent and geographically variable quality of care for people with dementia: in the UK, a Care Quality Commission found that “quality of care for people with dementia varies greatly and it is likely that they will experience poor care at some point along their care pathway” [8], and the London-based Health Foundation [9] found that examples of evidence-based guidelines and good practice in dementia care are inconsistently disseminated and implemented. In the US, the Dementia Action Alliance found that “dementia care in this country is impersonal and fragmented” [10] and the privately-funded Alzheimer’s Australia National Quality Dementia Care Initiative was explicitly established “to fast-track the implementation of existing dementia care research into wide-spread improvements in practice” [11].

The need for a better understanding of how to implement evidence-informed dementia care has also been recognised through priority setting partnerships and policy statements (e.g. James Lind Alliance/Alzheimer’s Society Dementia Priority Setting Partnership [12], Blackfriars Consensus on promoting brain health) [13]. In an attempt to identify and map the state of the evidence in implementation and dissemination in dementia care, we conducted a systematic scoping review of existing research in dissemination and implementation and used this to create a systematic evidence map. As such, our findings can be useful in prioritising areas of further implementation research in dementia care.

## Methods

Our scoping review was guided by the methods developed by Arksey and O’Malley [14, 15]. Scoping reviews provide an overview of the literature by mapping the key concepts in the evidence base of a research area and can be used to inform the need for a full systematic review and identify gaps in knowledge [14]. In contrast to systematic reviews, scoping reviews tend to have broader research questions to capture the range of evidence on the selected topic, apply inclusion and exclusion criteria that are often further developed and refined during the selection process, do not always involve detailed data extraction, and do not include an assessment of the methodological quality of included studies [15]. The aim of our scoping review was to systematically explore and describe the breadth and nature of available research in dissemination and implementation strategies within dementia care. We also wanted to identify the type of barriers and facilitators involved in the implementation process.

A project advisory group consisting of multiple stakeholders was established to work with the review team. The group involved carers and public with experience and interest in dementia care (Alzheimer’s Society research network), dementia friendly communities, communication, researchers and health professionals active in dementia care. The group met on three occasions and was involved in multiple stages of the project from the development of the review to the dissemination of findings. The methods for the scoping review were pre-specified in a protocol developed in collaboration with the project advisory group. The protocol was not registered with PROSPERO as scoping reviews do not fall within the remit of this initiative but is available from the authors on request.

### Study identification

A comprehensive search strategy was developed by an information specialist (MR) with input from the team using a combination of subject headings (MeSH terms) and free-text terms to cover the broad knowledge translation, implementation and dementia fields (Additional file 1). We undertook literature searches using the following databases from inception through October 2015: MEDLINE, Embase, PsycINFO, Healthcare Management Information Consortium (HMIC), Social Policy & Practice (SPP), Cochrane Database of Systematic Reviews (CDSR), Cochrane Central Register of Controlled Trails (CENTRAL), Cumulative Index to Nursing and Allied Health Literature (CINAHL), British Nursing Index (BNI), Applied Social Sciences Index and Abstracts (ASSIA), Social Sciences Citation Index (SSCI) and Conference Proceedings Citation Index (CPCI). We applied no language or methodological filters in searching. We subsequently searched citations of included papers (forwards citation searching) using Scopus and ISI Web of Science for potentially relevant studies. As an additional way of identifying grey literature we posted a request to CHAIN (Contact, Help, Advice and Information Network; an online mutual support network for people working in health and social care).

### Eligibility criteria

We included studies if they: (i) addressed dissemination or implementation strategies within dementia care or (ii) explored barriers and facilitators to dissemination or implementation and the strategies used to address them. For the purpose of this review, we used a definition of dissemination as ‘the targeted distribution of information and intervention materials to a specific public health or clinical practice audience, the intent of which is to spread knowledge’ [16]. We used a definition of implementation as ‘the use of strategies to introduce or change health and social care interventions within specific settings’ [16]. Dementia care refers to any aspect of health and social care support and services for people with dementia and their carers, in any setting. We included quantitative studies with a comparative research design and qualitative studies with recognised methods of data collection (e.g. interviews, focus groups) and synthesis (e.g. thematic or framework analysis, grounded theory). In order to be included, quantitative studies had to report on *implementation effectiveness,* i.e. the degree to which the implementation strategy of an innovation or intervention had been successful, rather than whether the intervention itself had been successful or effective. For example, studies aiming to improve the management of challenging behaviour in nursing homes through a new protocol had to report on the adherence to the protocol, and not simply on rates of change in challenging behaviour. Studies that included populations other than just people with dementia or populations with comorbid dementia were included if outcomes were reported separately for the sub-group with dementia. To capture the breadth of research in this area, we considered studies in care at all stages of dementia from first diagnosis through to palliative care and all settings of care. Populations of interest included people with dementia and those caring for them such as family caregivers, healthcare professionals, and other staff.

### Study selection

Titles and abstracts were screened for relevance independently by pairs of reviewers (IL and one of RA, JTC, MR, or IAL). Disagreements were resolved by discussion between reviewers or with the involvement of a third reviewer (RA, JTC, IAL) where necessary. We screened the full text of potentially relevant papers in the same way using the predefined inclusion and exclusion criteria. We had two non-English papers translated and contacted nine authors to request access to full-text reports. During the study selection process and as the team became more familiar with the nature of available literature, we refined and re-applied the initial criteria to reflect the focus of the question guiding the scoping review. Thus, we included studies exploring barriers and/or facilitators if they: (i) reported barriers/facilitators to the use of *identified* dissemination or implementation strategies (e.g. training, use of guidelines), (ii) related to a *change* in practice, knowledge or behaviour, or (iii) described experiences, perceptions, or attitudes towards the *use* of implementation strategies or *change* in practice, knowledge, or behaviour. We excluded studies that reported only barriers/facilitators and relevant experiences, perceptions, or attitudes to usual everyday care practices (i.e. not in the context of changing practice).

### Data charting

Data from the included studies were extracted and summarised by one reviewer (IL) and checked for accuracy by a second reviewer (RA) using bespoke forms developed in Excel. Disagreements were resolved by discussion. Extracted data included publication type, year and country, study design and methods, sample size, time frame, setting, topic area, target population, dementia stage, theory/framework used, details of the dissemination or implementation approach and relevant strategies, barriers and facilitators, and outcome variables.

We explored coding of dissemination and implementation approaches using two different classifications: the EPOC (Effective Practice and Organisation of Care) taxonomy of health systems interventions [17] and the ERIC (Expert Recommendations for Implementing Change) compilation of implementation strategies [18, 19]. The latest revised version of EPOC taxonomy organises complex health interventions into four main domains: delivery, financial and governance arrangements, and implementation strategies. Each domain contains categories and subcategories attempting to describe changes in how, when, and where healthcare is delivered, financial incentives and disincentives, rules and processes that may affect the organisation of services, and interventions or strategies that target healthcare professionals or organisations [17]. The ERIC compilation provides a summary of specific implementation strategies used to bring about change. ERIC aims to promote terminological consistency by organising a total of 73 distinct implementation strategies under nine thematic clusters. The clusters cover areas such as stakeholder training and education, clinician support, development of stakeholder interrelationships, changes in infrastructure, patient/consumer engagement, financial strategies, and the use of evaluative and iterative strategies to support practice change [19]. After testing both approaches in a small sample of papers (*n* = 12) and reflection in the review team, we decided the ERIC classification was more appropriate for this scoping review as it provides a more detailed and conceptually clear description of strategies. Included studies were coded independently by two reviewers (IL and RA) and are reported herein using the ERIC compilation. We charted data for the specific ERIC implementation strategies described in the studies and their allocated code (1–73) along with the corresponding cluster (1–9).

We adapted terminology from previous studies in knowledge translation interventions and contextual factors that may hinder or enable implementation [20–22] to classify barriers and facilitators within five categories: organisational, professional, individual, financial, other. Organisational factors relate to managerial and administrational support, the culture, organisation, management and facilities of settings providing dementia care. Professional factors relate to training, staff knowledge and skills. Individual factors include characteristics and attitudes of staff and other participants, and financial factors refer to operating costs and funding resources. We categorised outcomes as relating to staff members, to people with dementia, or to informal caregivers and family members with subcategories to reflect changes in practice (assessment, compliance, treatment, performance), knowledge, perceptions, behaviour, and physical health.

Consistent with the methods of scoping reviews, as described by Arksey and O’Malley [14, 15], we did not assess the methodological quality or risk of bias of included studies.

### Data analysis (mapping the evidence)

We tabulated and classified data according to the setting of dementia care provision and these are presented narratively below. We used tables (see Additional file 2: Table S1, Additional file 3: Table S2 and Additional file 4: Table S3), frequencies, and percentages to support narrative statements and provide an overview of the evidence base through summaries of the study characteristics (country, study design and methods, sample size, target population, topic area, broad category for focus of implementation, and context), implementation strategies, type of barriers/facilitators, and outcome type. We identified gaps in the literature during the process of collating and reporting the results using characteristics such as study design, topic area, setting, implementation-strategy cluster, related barriers and facilitators, and outcome.

## Results

### Literature search

**Fig. 1:**
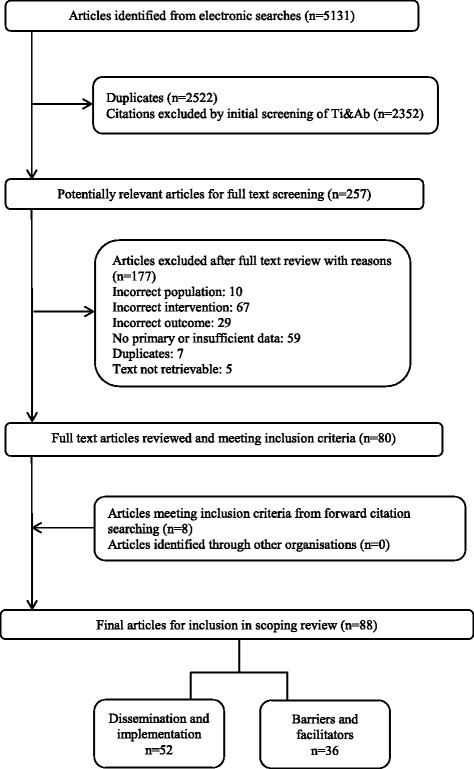
Flow chart of study selection process

Our electronic searches yielded 5131 citations. Deduplication and screening of titles and abstracts resulted in 257 papers for full text review of which 80 were eligible for inclusion. Our request to the CHAIN network resulted in the retrieval of 18 reports and studies of which none met the inclusion criteria. We identified eight additional papers through forward citation searching. In total, 88 papers met the inclusion criteria for this scoping review. The study flow with the number of identified citations, included studies, and reasons for exclusion is presented in Fig. 1. The full list of included studies is in Additional file 5.

### Study characteristics

Additional file 2: Table S1. presents a summary of the characteristics of all included studies sorted by setting of dementia care provision. Eighty-one of the included studies were peer-reviewed publications, two were dissertations, and five were independent reports. Publication year ranged from 1998 to 2015 but more than two-thirds of studies (69%, 61/88) were published in 2011 or later. The majority of studies were conducted in the USA (*n* = 22), followed by Australia (*n* = 18), the UK (*n* = 14), Canada (*n* = 12), Netherlands (*n* = 11), and other European (*n* = 6) and Asian countries (*n* = 3). One study collected data in England and the Netherlands [23] and another study included participants from nine European countries [24]. Thirty-nine percent of included studies were qualitative (34/88), 34% were quantitative (30/88) and 27% were mixed-methods studies (24/88).

A wide range of data collection methods were used across studies and in many studies multiple methods were used to collect data (e.g. cluster RCT plus interviews or focus groups plus surveys). Interviews were the most frequently reported study method (*n* = 25) followed by before/after studies (*n* = 19). Focus groups were used in ten studies, a combination of interviews and focus groups in 12 studies, and surveys in 14 studies. Eight studies were cluster RCTs, one was a RCT, and three were cohort studies. Other study designs and methods used included best practice implementation reports (i.e. JBI reports), quality improvement, and action research (*n* = 14). Reporting of implementation periods and of duration of follow-up was inconsistent. The wide variety of study methods and designs meant that studies described implementation activities ranging from half-day training to projects spanning a five-year period. Forty-nine percent (43/88) of studies reported follow-up data and the follow-up period ranged from one week to five years.

Nearly 60% (52/88) of the studies addressed dissemination and implementation interventions and the remainder (36/88) were concerned only with barriers/facilitators to dissemination or implementation activities without providing details or description of the implementation process. A combination of these (e.g. reporting of the implementation of a pain management protocol and also of barriers and facilitators to change) was reported in 26% of studies (23/88). The implementation strategies and the discussions around barriers and facilitators to change mostly targeted professionals: nursing staff (*n* = 27, 31%), care home and facility staff (*n* = 20, 23%), physicians (*n* = 11, 12.5%), other healthcare professionals (*n* = 11, 12.5%), and managers/leaders (*n* = 12, 14%). Other stakeholders actively involved in implementation initiatives across studies (*n* = 17, 19%) included researchers, experts in dementia care, activity therapists, psychologists, social-care workers, financial experts, police officers, architects, administrators, volunteers, and voluntary agencies. Relevant data for family members/caregivers involvement was included in 15% (13/88) of studies. Two studies sought the views of people with dementia. The dementia stage of residents and participants ranged from early through severe to end-of-life but it was unclear or not reported in 77% (68/88) of studies.

### Settings

We classified settings into five categories: residential long-term care, community care, primary care, hospital, and multiple. The residential long-term care category (*n* = 46) included care homes, nursing homes, assisted-living, skilled and residential aged care facilities, and dementia specialist-care units within homes or other long-term care facilities. Community care (*n* = 16) included studies taking place in non-residential care facilities and in the homes of people with dementia or caregivers. The primary care (*n* = 8) and hospital (*n* = 5) categories included studies explicitly stating those as the settings of the reported research. Multiple settings (*n* = 13) included studies in which dissemination or implementation activities took place in more than one of the above settings (e.g. nursing home and hospital). The number of participating or targeted sites across settings ranged from 1 to 15,453. About half of the studies were conducted within one site (52%, 46/88) and in 15 studies the number of sites ranged from two to ten (17%). Cluster RCTs (*n* = 8, 9%) included 9 to 45 sites.

### Focus of implementation

During data extraction we assigned a general descriptive theme to each included study. We then combined groups of studies that fitted conceptually together and, following reviewer agreement (IL and RA), we created a broad descriptive category. This process resulted in seven broad categories to describe the focus of included studies: Models of care (*n* = 17), Knowledge transfer and dementia education (*n* = 17), Behaviour management (*n* = 15), Care practices (*n* = 14), Guideline-driven practices (*n* = 12), Services and infrastructure (*n* = 8), and Care directives/frameworks (*n* = 5) (Additional File 2: Table S1). The ‘Models of care’ category included studies describing different models, methods and approaches to provide and improve dementia care such as person-centred care, capability model of care, and palliative approaches. The ‘Knowledge transfer and dementia education’ category included information exchange, use of research findings, factors influencing knowledge transfer, multifaceted implementation strategies, translation of caregiver intervention programmes, and other dementia training and outreach programmes for professionals. ‘Behaviour management’ included non-pharmacological and psychosocial interventions for BPSD, antipsychotic medication prescribing, and use of physical restraints. The ‘Care practices’ category included practices related to pain, oral health, bathing, sleep hygiene, mobility, and case management. The ‘Guideline-driven practices’ category included studies that examined the process or factors affecting the dissemination and implementation of specific guidelines. The development of memory clinics, meeting centres and other care units, the introduction of new services, evaluation of demonstration sites and facility design of residential settings were under the ‘Services and infrastructure’ category. The introduction and implementation of advanced care planning, Advance Directives, Do Not Hospitalise orders, and the Mental Capacity Act were represented in the ‘Care directives/frameworks’ category.

### Implementation and dissemination strategies

Of the 52 studies addressing dissemination and implementation within dementia care, five described dissemination, 36 described implementation and 11 reported both dissemination and implementation activities. The coding based on the ERIC compilation of implementation strategies is shown in Additional file 3: Table S2 and Additional File 4: Table S3. Although description of strategies was not always clear, we identified 55 out of the 73 strategies across all nine clusters. The majority of studies reported multifaceted implementation strategies which combine two or more discrete strategies but a few studies reported blended strategies which have been described as “multiple strategies packaged as a protocolized or branded implementation intervention” [25]. Studies reported a minimum of three and a maximum of 11 strategies covering between two and seven ERIC clusters. Additional File 3: Table S2 shows the total number of times each strategy was coded across the 52 studies. All but three studies had an educational component and used strategies described within the ‘Train and educate stakeholders’ cluster (*n* = 49, 94%). The most commonly reported strategy was educational meetings (*n* = 38) followed by the distribution of educational materials (*n* = 34) and dynamic training (*n* = 19). Strategies to develop stakeholder interrelationships (*n* = 36, 69%) and the use of evaluative and iterative strategies (*n* = 24, 46%) were frequently reported alongside training and educational strategies. Financial strategies were the least commonly reported (*n* = 8, 15%). Eleven strategies across clusters were reported once and 18 of the ERIC strategies were not identified at all (Additional File 3: Table S2).

**Fig. 2:**
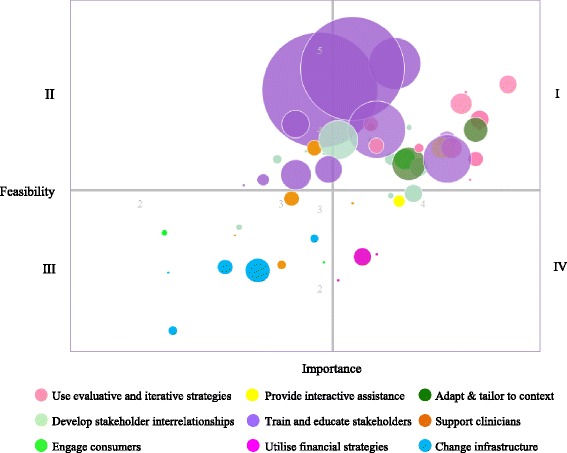
Bubble plot for the frequency of the 55 implementation strategies identified within included studies based on ERIC importance and feasibility ratings. The range of the x and y axes reflect values obtained for the 73 discrete implementation strategies for each of the rating scales during the ERIC rating tasks.^18^ The plot is divided into quadrants on the basis of the overall mean values for each of the rating scales. Strategies in quadrant I are those with the highest consensus regarding their relative high importance and feasibility. Strategies in quadrant III are those where there was consensus regarding their relative low importance and feasibility. Strategies in quadrant II were relatively high in feasibility but low in importance, and strategies in quadrant IV were relatively high in importance but low in the feasibility scale

Waltz and colleagues [19] present in their paper a graphical summary of the 73 ERIC implementation strategies based on their mean importance and feasibility ratings as determined by expert consensus. The majority of strategies in the high importance and high feasibility category lie within clusters 1, 4 and 5 which are also among the most identified strategies in our data (pink, light green and purple respectively, quadrant I; Fig. 2). Nevertheless, the individual highly important and feasible implementation strategies within these clusters have not been reported frequently across the reviewed studies (e.g. strategies #4,#5,#18,#33,#38; Additional File 3: Table S2). Financial strategies generally received a low feasibility rating and we found only a few studies reporting these (dark pink, quadrant IV).

### Outcome characteristics

Nearly half of the studies (*n* = 47, 53%) reported staff-related outcomes and within these studies 29% (*n* = 14) also reported outcomes related to people with dementia and family/caregivers (Additional File 4: Table S3.). Forty-five studies used some quantitative measure of effectiveness linked to implementation as the staff-related outcome. The most commonly used outcome that also reflected a measurement of effectiveness was compliance (e.g. compliance with guidelines or use of tool, *n* = 28), followed by change in knowledge (*n* = 17), and perceptions and attitudes (*n* = 14). Change in behaviour (e.g. agitation) was the most frequent outcome for people with dementia (*n* = 8), while perceptions were most frequently investigated within family/caregiver outcomes (*n* = 4). Overall, significant changes in practice, knowledge or perceptions/attitudes were reported in 23 of the 47 studies (49%). The majority of both staff-related (*n* = 29) and non-professional (*n* = 8) outcomes were studied in the residential long-term care settings.

### Barriers and facilitators

Studies reported collecting data on barriers and/or facilitators mostly using interviews and focus groups but also as part of surveys, questionnaires, and field notes. Barriers and facilitators were reported in 67% (59/88) of studies in total (implementation strategies plus barriers/facilitators *n* = 23, only barriers/facilitators *n* = 36). More specifically, 22 studies focused solely on barriers, four studies focused solely on facilitators, and a combination of hindering and enabling factors was reported in 33 studies. The dominant factor was organisational, highlighted in 91% of studies on barriers and facilitators (54/59) (Additional File 4: Table S3). Time constraints, increased workload, leadership, and managerial support were common themes in this category. Professional factors were identified in 52% of studies (31/59) and included lack of dementia-related knowledge, training and experience using tools, and behavioural strategies. Personality characteristics of staff members, engagement, resistance to change, and other individual factors were reported in 51% of studies (30/59). Financial factors such as lack of funding or financial constraints were reported in 15 studies. Other identified barriers were environmental (physical structure limitations), legal (boundaries and legal status of advance care planning), resident-specific (poor health status), and dementia-specific (cognitive impairment and other complications in the course of the disease) factors (each reported once).

### Use of frameworks

Thirty-eight studies reported using a theory or framework as part of the implementation process (43%, 38/88). A total of 33 different frameworks were reported. RE-AIM [26] was the most commonly cited framework (*n* = 5) and the Joanna Briggs Institute PACES and GRIP programme [27] was the most frequently used online tool used to conduct audits and facilitate practice change (*n* = 5). Four studies referred to frameworks that addressed the dissemination stage of the intervention (Train-the-trainer model, Kerr and Slocum’s model of performance, Diffusion of Innovation Theory) but frameworks were generally used to guide or evaluate the wider implementation process and this usually also included dissemination. Twelve studies used a theory or framework to inform the identification and description of barriers and facilitators to dissemination (*n* = 2) and implementation (*n* = 10).

### Implementation stages

The numerous theories and frameworks available [28] to inform and enhance implementation research highlight the dynamic nature of this process, which is usually characterised in several stages. According to the EPIS conceptual model [29] there are four phases of implementation: Exploration, Preparation, Implementation, and Sustainment. The majority of the studies we included focused on the implementation phase either in terms of strategies used for change or related barriers and facilitators. There was little attention to the Exploration phase, (*n* = 3) where potential implementers searched the literature for evidence-based practices to suit their needs and/or assessed readiness for change. Few studies described a preparation stage (*n* = 6) that includes assessment of implementation challenges, and all best practice implementation projects involved a planning stage following an initial audit (*n* = 6). Thirty studies addressed some aspect of Sustainment: (i) ten studies measured the sustainability of a project or included a relevant maintenance stage and outcome (e.g. studies describing the maintenance stage as part of the RE-AIM framework), and (ii) 20 studies described factors affecting project sustainability or reported plans and suggestions to maintain project implementation.

## Discussion

In this scoping review we identified 88 primary studies addressing dissemination and implementation research across various settings of dementia care published between 1998 and 2015. Our findings indicate a paucity of research focusing specifically on dissemination of knowledge within dementia care and a limited number of studies on implementation in this area. We also found that training and education of professionals, development of stakeholder interrelationships and the use of evaluative and iterative strategies are frequently employed to introduce and promote change in practice. However, although important and feasible, these strategies only partly address what is repeatedly highlighted in the evidence base: that organisational factors are reported as the main barrier to implementation of knowledge within dementia care [30–35]. Moreover, included studies clearly support an increased effort to improve the quality of dementia care provided in residential settings in the last decade. Nevertheless, people with dementia and their family members have been rarely involved in implementation research and their views and experiences have generally not been considered as part of implementation process [36, 37]. Funding for dementia research has increased markedly in the past decade and this has led to an increase in research outputs. However, assuming that increased levels of research will lead to changes in practice, perhaps based on some poorly-conceived notions of knowledge diffusion, is at best naïve and at worst recklessly wasteful. That we found so few papers on dissemination and implementation in dementia care is a sign that this aspect of improving quality of dementia care has been neglected and is in urgent need of greater attention and more resources, as has been previously highlighted [12, 13]. Without this, even the best dementia research will go to waste, in which case everybody – funders, researchers, and people affected by dementia – loses out.

Health services research in other areas of care suggests that implementation strategies to promote evidence-based practice and improve quality of care are dominated by educational approaches to train professionals mainly through educational meetings and the distribution of educational materials, reminder systems to facilitate clinician decision-making, and evaluative strategies such as audit and feedback [38]. Our findings reveal a similar picture for implementation in the field of dementia care. Synthesised evidence in guideline implementation research indicates that although most implementation strategies result in small to moderate improvements in quality of care, there is an increased likelihood of positive results in practice, knowledge or patient outcomes with the use of multifaceted interventions that also target barriers to change and actively engage stakeholders [38–40]. Multiple strategies have been reported within individual studies in this review and our findings show that educational strategies are often combined with organisational-level approaches to support stakeholder interrelationships, evaluative and iterative strategies, and occasionally changes in infrastructure as part of practice change in dementia care. However, only a small proportion of studies reported a stage in the implementation process dedicated to the identification of barriers and facilitators or strategies tailored to address them. In addition, 30% of the ERIC compilation strategies generated by expert consensus to guide implementation were not identified in the included studies. Many of these strategies describe financial approaches (e.g. access new funding, use capitated payments) where there was consensus regarding their relative high importance but low feasibility [19]. Although it is unlikely that this finding is unique to dementia care, the degree to which these specific strategies could promote implementation within the field remains to be investigated. The usefulness of the ERIC compilation to characterise implementation strategies within dementia care that are also relevant to health care systems other than in the US should be explored further.

The majority of included studies covering barriers and facilitators to implementation reported some factor lying at the organisational level. This finding occurred across settings and was particularly prominent among nurses and other care staff. Frequent reports highlighting the role of managerial support and insufficient time to complete heavy workload are consistent with evidence on organisational culture factors that act respectively as facilitators of and barriers to implementation of best practice from different healthcare disciplines [41, 42]. It seems reasonable to suggest that comprehensive approaches with strategies tailored to promote identified organisational facilitators and overcome barriers in dementia care would promote practice change. However, studies on the effectiveness of such strategies are limited [43]. Professional factors and individual characteristics identified in our scoping review including lack of dementia-specific knowledge, resistance to change, held attitudes, staff engagement and competence also appear to play a role in the implementation of dementia care practices [32, 44–48]. This indicates that future implementation efforts would benefit from a preparation stage to identify potential barriers and facilitators, and subsequently plan for multifaceted strategies that address the different levels informed by the needs and desires of relevant stakeholders in a constantly changing environment of care provision. Additional research using theories/models to identify and describe the various barriers and facilitators of desired change [21] at the micro-, meso- and macro-level is needed to shed light on the key predictors of change and the complex dynamics of implementation content, enabling and hindering factors within dementia care.

Much of the available literature covers research conducted within residential settings predominantly nursing and care homes. As such, many studies were identified in areas of care including behaviour assessment and management and models of care targeting people with dementia that are of particular interest in these settings. They reflect the great challenges nursing staff and other healthcare professionals face in managing symptoms as well as efforts for quality improvement in residential care facilities internationally [49, 50]. However, there is very limited evidence relating to implementation of strategies for initiatives to manage comorbidities in people with dementia [51] within long-term care facilities, and an evidence gap in translating research into practice in terms of transitions between care settings [52]. The review also suggests that published research in implementation efforts to improve dementia care practice in hospitals and primary care clinics does not match the increasing demand of these settings to care for people with dementia and their caregivers [53]. In addition, little is known about how best to put practices into action to support family caregivers of people with dementia living in the community or the implementation of dementia care practices at the end of life. As evidence grows, these areas should also be prioritised as implementation targets to promote high-quality dementia care and deliver on the ‘living well with dementia’ challenge [54].

Although we did not conduct a formal quality assessment of the included studies, we identified a few limitations. Most of the studies did not report the dementia characteristics (e.g. type and severity) of populations, which should be included in future studies. The duration of implementation was unclear in many studies and it was often difficult to differentiate between the implementation period and duration of follow up. Such characteristics of the condition and context are crucial in order to map the extent and nature of implementation research across the dementia care pathway, and to illuminate areas of care for knowledge translation that may be particularly relevant to certain stages of the dementia journey. The coding of implementation strategies was also challenging in many situations due to inadequate reporting of the activities employed for implementation. This lack of clarity adds to the challenging task of distinguishing between implementation of strategies and implementation of interventions due to overlaps in terminology and interpretation. Overall, there is a need for better reporting of implementation research to promote study identification, increase transparency and replicability, and improve the evaluation of studies.

### Strengths and limitations

This is the first scoping review of dissemination and implementation research within dementia care. Previous research on implementation of evidence-based practice has investigated knowledge translation interventions and contextual factors in health care settings across various chronic conditions but little research has examined the implementation strategies used to promote best practice and the associated barriers and facilitators in dementia care. Our scoping review presents the extent and nature of current literature on efforts to translate research and change practice in dementia care and what is known about the factors that may enable or hinder this process. Our review builds on the evidence base from a number of systematic reviews that have addressed discrete areas of improving dementia care [55–59]. Whilst mostly concerned with effectiveness of interventions [55, 56, 59], these reviews have addressed some elements of dissemination and implementation. Elliot [56] and Reis [58] highlight the lack of detail reported on implementation in their reviews of training interventions. Reis [58] and Spector [59] emphasise the limited accessibility and lack of reporting on training manuals which impact the ability to reproduce interventions. Perry [55] and Eggenberger [57] concluded that education as a means to bring about change worked better when supported with another strategy – either financial or some form of feedback. Our scoping review shows that across dementia care settings and topics, there is a commonality of issues for dissemination and implementation that are yet to be resolved.

While we performed comprehensive searches across the most relevant databases and conducted forward citation searches of the included studies, we did not review their reference lists or hand-searched relevant journals due to the large number of studies. We sought to identify and have included unpublished research in our study. However, the difficulty of searching the grey literature may have thwarted our attempt to identify relevant unpublished material. Additionally, implementation research is a growing field with multiple terms to describe dissemination and implementation [60] so it is possible we may have missed some relevant articles. However, our search strategy and study selection process followed systematic review methods and we are confident that this scoping review provides a representative range of the implementation literature in dementia care. The scoping nature of the review precluded the detailed description of implementation characteristics across care settings, and the assessment of quality and effectiveness of strategies of included studies. As such, we are not able to provide recommendations for the implementation of specific strategies to promote practice change within dementia care settings. However, this review has informed the feasibility of a full systematic review and we plan to evaluate the effectiveness of implementation strategies on process outcomes across the various settings of dementia care provision.

## Conclusions

This scoping review and systematic mapping of the evidence reveals a paucity of robust evidence to inform the successful dissemination and implementation of evidence-based dementia care. Noteworthy gaps in the evidence include research to inform effective methods of dissemination and implementation in hospital and primary care settings, and to support people with dementia and their carers living in the community. On the whole, the reporting of implementation strategies is poor with insufficient detail to enable replication. Further exploration of the most appropriate methods to evaluate and report initiatives to bring about change across settings and of the effectiveness of implementation strategies is necessary if we are to make changes in practice that improve dementia care.

## Additional files

Additional file 1: MEDLINE search strategy. (PDF 90 kb)

Additional file 2: Table S1. Characteristics of included studies categorised by care setting. (DOCX 36 kb)

Additional file 3: Table S2. Implementation strategies identified across studies (*n* = 52) coded based on the ERIC compilation. (DOCX 19 kb)

Additional file 4: Table S3. Summary of implementation strategies and outcomes across studies categorised by care setting. (DOCX 24 kb)

Additional file 5: List of studies included in the systematic scoping review (*n* = 88). (PDF 174 kb)

## Abbreviations

ASSIA: Applied social sciences index and abstracts
BNI: British nursing index
CDSR: Cochrane database of systematic reviews
CENTRAL: Cochrane central register of controlled trails
CHAIN: Contact, help, advice and information network
CINAHL: Cumulative index to nursing and allied health literature
CPCI: Conference proceedings citation index
EPIS: Exploration, preparation, implementation, sustainment model
EPOC: Effective practice and organisation of care
ERIC: Expert recommendations for implementing change
HMCI: Healthcare management information consortium
JBI: Joanna Briggs Institute
MeSH: Medical subject headings
RCT: Randomised controlled trial
RE-AIM: Reach effectiveness adoption implementation maintenance framework
SPP: Social policy & practice
SSCI: Social sciences citation index
