# Supplementary material for: Dissemination and implementation research in dementia care: a systematic scoping review and evidence map
Source: BMC Geriatr. 2017 Jul 14;17:147. doi: 10.1186/s12877-017-0528-y (PMC5513053; doi:10.1186/s12877-017-0528-y)
Supplement: Supplementary file 1 — MEDLINE search strategy. (PDF 90 kb) [file 12877_2017_528_MOESM1_ESM.pdf]

Appendix A

MEDLINE search strategy

Database: Ovid MEDLINE(R) In-Process & Other Non-Indexed Citations and Ovid MEDLINE(R) <1946 to Present>
Search Strategy:
----

1 Dementia/co, et, mo, nu, pc, px, rh, th [Complications, Etiology, Mortality, Nursing, Prevention & Control, Psychology, Rehabilitation, Therapy] (22834)
2 Alzheimer Disease/co, et, mo, nu, pc, px, rh, th [Complications, Etiology, Mortality, Nursing, Prevention & Control, Psychology, Rehabilitation, Therapy] (25312)
3 dementia.ti,ab. (74081)
4 alzheimer*.ti,ab. (102711)
5 or/1-4 (156988)
6 exp Nursing Care/ (120786)
7 exp Residential Facilities/ (45050)
8 Health Services for the Aged/ (15623)
9 care.ti,ab. (972256)
10 (hospital* or ward*).ti,ab. (935325)
11 hospice*.ti,ab. (9119)
12 carer*.ti,ab. (9122)
13 healthcare.ti,ab. (113400)
14 caregiver*.ti,ab. (36916)
15 communit*.ti,ab. (374747)
16 (home or homes).ti,ab. (179162)
17 primary care.ti,ab. (79330)
18 general practice.ti,ab. (30346)
19 outpatient*.ti,ab. (122086)
20 (clinic or clinics).ti,ab. (225824)
21 (pharmacy or pharmacies or chemist or chemists).ti,ab. (38021)
22 schools.ti,ab. (58486)
23 ((GP* or doctor*) adj (surger* or practice*)).ti,ab . (1494)
24 or/6-23 (2439543)
25 Health Plan Implementation/ (4276)
26 information dissemination/ (11811)
27 exp "diffusion of innovation"/ (16545)
28 action research.ti,ab. (2544)
29 healthcare innovation.ti,ab. (48)
30 "bench to bedside".ti,ab. (2080)
31 "barriers and facilitators".ti,ab. (2165)
32 (barriers and facilitators).ti. (943)
33 (translational adj (medicine or science or research)).ti,ab. (6871)
34 (information adj3 dissemination).ti,ab. (1877)
35 knowledge adoption.ti,ab. (8)
36 (knowledge adj (brokering or communication)).ti,ab. (134)
37 (knowledge adj (cycle or development or application)).ti,ab. (563)
38 (knowledge adj (diffusion or exchange)).ti,ab. (332)
39 (knowledge adj (mobili*ation or synthesis)).ti,ab. (142)
40 (knowledge adj (transfer or translation or transformation)).ti,ab. (2204)
41 (knowledge adj (update or utili*ation)).ti,ab. (110)
42 "know do gap".ti,ab. (46)
43 integrated knowledge.ti,ab. (230)
44 integrating knowledge.ti,ab. (140)
45 "knowledge to action".ti,ab. (437)

46 "linkage and exchange".ti,ab. (22)
47 organi?ational innovation.ti,ab. (63)
48 technology transfer.ti,ab. (813)
49 (translational adj (medicine or research or science)).ti,ab. (6871)
50 "transmission of knowledge".ti,ab. (245)
51 "research into practice".ti,ab. (620)
52 (research adj2 integration).ti,ab. (444)
53 (research adj2 utili?ation).ti,ab. (1030)
54 (implementation or implementing).ti,ab. (172581)
55 (dissemination or disseminating).ti,ab. (41672)
56 (transfer* adj2 knowledge).ti,ab. (1692)
57 "barriers and facilitators".ti,ab. (2165)
58 sustainability.ti,ab. (10505)
59 ((change or changing) adj (behavio?r or practice)).ti,ab. (2017)
60 or/25-59 (262780)
61 5 and 24 and 60 (1083)
