# Supplementary material for: Dissemination and implementation research in dementia care: a systematic scoping review and evidence map
Source: BMC Geriatr. 2017 Jul 14;17:147. doi: 10.1186/s12877-017-0528-y (PMC5513053; doi:10.1186/s12877-017-0528-y)
Supplement: Supplementary file 2 — Characteristics of included studies categorised by care setting. (DOCX 36 kb) [file 12877_2017_528_MOESM2_ESM.docx]

| **Table S1. Characteristics of included studies by care setting** | | | | | | | | |  |  |  |
| --- | --- | --- | --- | --- | --- | --- | --- | --- | --- | --- | --- |
| **First author (year)** | **Country** | **Study type** | **Size** | **Target population** | **Study topic area** | **Broad category of study focus** | **Dissemination/**  **Implementation/**  **Both** | **Context/Overall aim** |  |  |  |
| **Residential long-term care (n=46)** | | | | | | | | |  |  |  |
| Andrews (2009) | Australia | Action research,  Mixed (interviews, survey) | 15 ppts | Nurses, unregulated workers, family caregivers | Guideline-driven palliative approach to care | Guideline-driven practices | Both | Action research project investigating possibilities for residential aged care facility staff to develop their practice to support an evidence-based palliative approach to care provision |  |  |  |
| Aveyard (2006) | UK | Action research, Qualitative (interviews, group discussion) | 18 ppts | Staff, relatives of PwD | Collaborative working for resident-centred care | Models of care | NA | To describe the evaluation of an action group project aimed at improving experiences of living and working within the nursing home underpinned by the Senses Framework |  |  |  |
| Brooker (2014) | UK | Report,  Mixed (before/after study, interviews) | 67 CHs,  66 Dementia Care Coaches | Managers, nurses, care home trainers | Translation of programme to reduce the use of antipsychotic medication | Behaviour management | Implementation | To summarise the evaluation of the impact and process of the FITS into Practice intervention implemented in care homes and describe the barriers and facilitators to its implementation |  |  |  |
| Burgio (2002) | USA | Quantitative  (cluster RCT) | 9 nursing units,  85 staff | Certified nursing assistants (CNAs) | Behaviour management skills programme | Behaviour management | Implementation | To examine the efficacy of a behaviour management skills training programme for improving CNAs skill performance in the nursing home and to assess the effectiveness of a staff motivation system for maintaining newly acquired skills |  |  |  |
| Calleson (2006) | USA | Quantitative  (surveys, cross-sectional user data) | 15,453 NHs | Nurses, administrators | Bathing educational programme | Care practices | Dissemination | To describe the development and distribution of an educational programme for nursing home staff, and the evaluation of its dissemination and use |  |  |  |
| Chenoweth (2015) | Australia | Qualitative  (interviews, telephone surveys, field notes) | 172 ppts | Managers, nurses and care staff, family members | Person-centred care | Models of care | NA | To identify views on barriers and facilitators to the implementation of person-centred care interventions in long-term care in the PerCEN trial |  |  |  |
| Cooke (2014) | Australia | Mixed  (surveys, field notes) | 48 ppts | Nurses, personal care workers, other staff | Capacity model of dementia care | Models of care | Implementation | To evaluate an educational intervention implementing the capability model of dementia care and explore perceptions of the challenges involved in practice change |  |  |  |
| Dickson (2015) | USA | Quality improvement project, Quantitative  (before/after study) | 20 staff,  10 residents | Nursing staff | Pain assessment and management | Care practices | Implementation | Describes a quality improvement project for pain assessment and management at an assisted-living facility guided by the Plan-Do-Study-Act cycle process |  |  |  |
| Fallon (2006) | Australia | Quality improvement project,  Mixed (before/after survey, focus groups) | 2 facilities, 50 staff | Care home staff | Oral hygiene practices | Care practices | Implementation | Quality improvement project aiming to introduce published oral hygiene practices in 2 residential aged care facilities, monitor for changes in knowledge and practice and identify barriers to implementation using the Plan-Do-Check-Act approach |  |  |  |
| Finucane (2013) | UK | Sustainability project,  Quantitative  (before/after study) | 7 CHs | Care home staff | Organisation of palliative care | Models of care | Implementation | Describes a sustainability project designed to sustain the results following the implementation of a programme to improve care to residents approaching end of life through better organisation of care |  |  |  |
| Fleming (2012) | Australia | Mixed  (cross-sectional observation, interviews) | 10 facilities | Senior managers, architects | Facility design | Services and infrastructure | NA | To explore the obstacles to the application of existing knowledge on good design of aged care facilities for PwD using the Pathman’s model of knowledge translation |  |  |  |
| Gnaedinger (2003) | Canada | Qualitative  (interviews, focus groups, diary notes) | 38 ppts | Practice leaders, front line workers | Resident-centred model of care | Models of care | NA | Describes experiences of barriers and facilitators to the implementation of resident-centred dementia care in long-term care settings |  |  |  |
| Hoffman (1998) | USA | Mixed  (survey) | 77ppts | Various staff | Dementia care programme challenges | Knowledge transfer and dementia education | NA | Survey to identify problems encountered in the implementation of dementia care programmes and strategies to address them |  |  |  |
| Holle (2015) | Germany | Quasi-experimental (interviews, observational field notes) | 12 NHs,  18 nursing teams | Nursing staff | Assessment and management of BPSD | Behaviour management | NA | To explore experiences of nursing staff using dementia-specific case conferences with the Innovative dementia oriented assessment tool and the contextual barriers and facilitators to its implementation |  |  |  |
| Hynes (2014) | Australia | Best practice implementation project, Mixed (before/after study, group discussion) | 80 staff, 17 residents | Care staff | Assessment and management of BPSD | Behaviour management | Implementation | Reports on a project to promote evidence-based assessment and non-pharmacological management of challenging behaviour in a residential setting |  |  |  |
| Janzen (2013) | Canada | Qualitative  (interviews, focus groups, questionnaire) | 5 facilities, 44 ppts | LTC staff | Non-pharmacological interventions for agitation management | Behaviour management | NA | To investigate staff’s perceptions regarding use of non-pharmacological interventions for reducing agitation and to identify barriers and facilitators to implementation in the long-term care facilities |  |  |  |
| Kolanowski (2015) | USA | Qualitative  (focus groups) | 2 NHs,  59 ppts | Nurses and other NH staff | Person-centred care for BPSD | Models of care | NA | To investigate barriers to information exchange and implementation of person-centred care for nursing home staff |  |  |  |
| Kovach (2008) | USA | Mixed  (survey, log records) | 9 NHs,  14 ppts | Nurses | Pain assessment and management | Care practices | Both | Describes the implementation of a tool to improve pain assessment and management in nursing homes guided by the Diffusion of Innovation Theory principles and barriers and facilitators to this process |  |  |  |
| Lawrence (2015) | UK | Qualitative  (focus groups) | 16 CHs,  119 ppts | Care home staff | Psychosocial interventions | Behaviour management | NA | To explore attitudes, expectations, challenges and priorities of care home staff regarding a new training programme to deliver psychosocial interventions |  |  |  |
| Mann (2013) | USA | Qualitative  (interviews) | 16 ppts | Healthcare proxies | Do-Not-Hospitalise orders | Care directives/frameworks | NA | To explore how healthcare proxies for nursing home residents with advanced dementia make decisions about DNH orders and why they may or may not initiate them |  |  |  |
| Mellor (2015) | Australia | Quantitative  (before/after study) | 99 staff,  101 residents | Personal care assistants, registered nurses, other staff | Protocol for BPSD management | Behaviour management | Implementation | To investigate whether external clinical support to implement a structured protocol for BPSD management improved compliance in its use over and above only providing a generic workshop |  |  |  |
| Monette (2008) | Canada | Quantitative  (prospective observational pilot study) | 203 staff,  90 residents | Physicians, pharmacists, nursing home staff | Reduction of antipsychotic medication | Behaviour management | Both | To assess the effect of an interdisciplinary educational programme in reducing the use of antipsychotics in nursing home residents |  |  |  |
| Morgan (2005) | Canada | Qualitative  (interviews) | 10 ppts | Nursing directors | Development and sustainment of dementia Special Care Units (SCUs) | Services and infrastructure | NA | Describes the development of SCUs in small rural nursing homes and barriers and facilitators to their success and sustainment |  |  |  |
| Moyle (2013) | Australia | Qualitative  (focus groups, interviews) | 25 ppts | Various staff, family members, residents | Capabilities model of dementia care | Models of care | Implementation | To assess the experience of the capabilities model of care implementation with reference to the process, outcomes and barriers and facilitators |  |  |  |
| Munir (2007) | USA | Quality improvement project,  Quantitative  (retrospective chart review, before/after study) | Number of staff involved not reported,  83 residents | Physicians, pharmacists | Calcium and vitamin D supplementation | Care practices | Implementation | To evaluate the impact of a quality improvement study on improving calcium and vitamin D supplementation in a long-term care setting |  |  |  |
| Murray (2011) | Australia | Best practice implementation project,  Quantitative (before/after study) | 30 staff,  30 residents | Nursing staff | Physical restraint minimisation | Behaviour management | Implementation | Reports on a project to promote minimisation of physical restraints in a secure dementia unit |  |  |  |
| Oye (2015) | Norway | Qualitative (ethnographic study) | 3 NHs,  30 staff | Care staff | Prevention of restraint use | Behaviour management | Implementation | Post-intervention ethnographic study to investigate the contextual factors influencing knowledge utilisation of an education intervention in nursing homes guided by the PARIHS framework |  |  |  |
| Ranasinghe  (2013) | Australia | Best practice implementation project,  Quantitative (before/after study) | 80 staff,  30 residents | Care staff | Clinical risk assessment and management | Care practices | Implementation | Reports on a project to promote risk assessment using evidence-based tools for care planning when caring for residents with severe dementia |  |  |  |
| Rapp (2013) | Germany | Quantitative  (cluster RCT) | 18 NHs,  304 residents | Nursing staff | Guidelines for management of behavioural symptoms | Guideline-driven practices | Implementation | To evaluate the effect of a complex-guideline implementation on agitation and psychotropic prescriptions in nursing homes |  |  |  |
| Roberts  (2015) | Australia | Quality improvement project,  Mixed (before/after study, interviews) | 18 staff,  16 residents, 15 families | Nursing,  care and environmental services staff | ABLE model of care | Models of care | Implementation | To describe the development of a composite model of care in a residential care unit and its impact on PwD, family members and staff |  |  |  |
| Rokstad (2015) | Norway | Qualitative  (focus groups) | 25 ppts | Nursing staff and leaders | Leadership in person-centred care | Models of care | NA | To investigate the role of leadership types in the implementation of person-centred care using Dementia Care mapping in nursing homes |  |  |  |
| Rooney (2014) | Australia | Best practice implementation project,  Quantitative  (before/after study) | 29 staff,  18 residents | Registered nurses, care staff | Guidelines for management of behavioural symptoms | Guideline-driven practices | Implementation | Reports on a project to promote best practice strategies to assess, manage and reduce physical aggression and improve resident and staff outcomes |  |  |  |
| Sacoco (2014) | USA | Best practice implementation,  Quantitative  (before/after study) | 33 staff,  27 residents | Nurses | Pain assessment | Care practices | Implementation | To describe the process of seeking the best evidence-based practice for pain assessment and its implementation in nursing home residents |  |  |  |
| Sidani (2009) | Canada | Quantitative  (before/after study) | 79 ppts | Nursing staff | Abilities-focused approach to care | Models of care | Implementation | To determine the type and number of abilities-focused interventions implemented by nursing staff to manage agitation in residents during morning care |  |  |  |
| Simpson (2007) | USA | Qualitative (interviews) | 8 ppts | Nurses | Pain assessment | Care practices | NA | To describe nurses’ experience, barriers and facilitators to the clinical application of a protocol for pain assessment within a long-term care setting |  |  |  |
| Slaughter (2013) | Canada | Quasi-experimental (before/after, interview-based survey) | 2 NHs,  56/71 staff,  26 residents | Healthcare aides | Mobility innovation | Care practices | Implementation | To assess the effect of a mobility activity, the method of facilitation and contextual factors influencing its uptake by healthcare aides in nursing homes |  |  |  |
| Sung (2008) | Taiwan | Quantitative  (before/after study) | 17 ppts | Nursing staff | Music protocol for agitation management | Behaviour management | Both | To evaluate the effects of an implementation programme on nursing staff’s knowledge of and adherence to an individualised music protocol for agitation management of PwD |  |  |  |
| Teri (2009) | USA | Qualitative  (interviews, observation forms, field notes) | 8 facilities, 80 staff,  36 residents | Leadership staff , assistive personnel | Dementia care training programme | Knowledge transfer and dementia education | Both | Describes the provision of the STAR training programme to assisted living residences, challenges in its implementation and how they were addressed |  |  |  |
| Timmins (2008) | Australia | Best practice implementation project,  Quantitative  (before/after study) | 73 residents | Nurses, personal care staff | Physical restraint minimisation | Behaviour management | Implementation | Reports on a project to improve clinical practice in the use of physical restraint by auditing compliance with best practice criteria in a residential care facility |  |  |  |
| Tjia (2015) | USA | Mixed  (cluster RCT, interviews) | 42 NHs,  302 staff | Leaders and care staff | Guidelines for reduction of antipsychotic medication | Guideline-driven practices | Both | To evaluate the effectiveness of strategies to translate evidence-based guidelines about antipsychotic medication use to nursing homes using the RE-AIM framework |  |  |  |
| Van der Kooij (2013) | Netherlands | Quantitative  (cluster RCT) | 14 NHs,  124 staff | Care staff | Psychosocial care approach | Models of care | Implementation | To evaluate the effectiveness of the implementation of the ‘integrated emotion-oriented care’ approach in nursing homes |  |  |  |
| Van Haeften  (2015a) | Netherlands | Process evaluation, Qualitative  (focus groups, interviews) | 12 stakeholders,35 staff | Mostly activity therapists | Communication method | Models of care | Implementation | To evaluate the implementation of the Veder communication method on nursing home wards while describing the barriers and facilitators of the process using the Implementation Process Evaluation Framework |  |  |  |
| Verkaik (2011) | Netherlands | Mixed  (interviews, reports, observations, scores) | 9 NHs,  98 staff | Certified nursing assistants (CNAs) | Nursing guideline on depression in dementia | Guideline-driven practices | Implementation | To assess the level of success of the guideline introduction in nursing home wards and describe the facilitating and inhibiting factors influencing the introduction and application of the guideline |  |  |  |
| Vida (2012) | Canada | Quantitative  (before/after study) | 46 residents | Physicians, nurses | Reduction of antipsychotic medication | Behaviour management | Implementation | To assess rates of antipsychotic medication use 5 years after the first intervention and to determine whether use could be further reduced by implementing an updated programme |  |  |  |
| Vikstrom (2015) | Sweden | Action research, Qualitative (process notes, experiential data) | 200 ppts | Managers, nursing staff | Guidelines for dementia care | Guideline-driven practices | Implementation | To describe a model for implementing national, evidence-based guidelines for care of PwD and staff’s experiences with the implementation process following a participatory action research approach |  |  |  |
| Zwijsen (2014) | Netherlands | Process evaluation,  Mixed  (questionnaires, interviews) | 17 NHs,  64 ppts | Nursing staff, physicians, psychologists | Management of challenging behaviour | Behaviour management | NA | To evaluate the process and describe barriers and facilitators to implementation of a care programme for managing challenging behaviour in the dementia special care units of nursing homes |  |  |  |
| **Hospitals (n=5)** | | | | | | | | |  |  |  |
| Atkinson (2012) | UK | Mixed  (focus groups, quant) | 1 hospital | Nurses | Nurse-lead liaison service | Services and infrastructure | Implementation | To describe the improvement process and implementation of a liaison service for older adults based on the Toyota Production System |  |  |  |
| Banks (2014) | UK | Mixed  (before/after study, evaluation survey) | Various acute settings,  93 ppts | Various HCPs inc. nurses | Dementia Champions training program | Knowledge transfer and dementia education | Implementation | Describes the development, delivery and evaluation of a training programme to prepare Dementia Champions working in acute settings as Change Agents for practice |  |  |  |
| Henderson (2006) | Australia | Quasi experimental  (before/after survey) | 1 hospital,  39/38 ppts | Mostly nurses | Use of research findings | Knowledge transfer and dementia education | Implementation | To assess the impact of educational strategies introducing research evidence on awareness and inclination to use research findings in future practice |  |  |  |
| Luxford (2015) | Australia | Mixed  (time series analysis, surveys) | 21 hospitals, 128 clinicians, 240 carers | Clinicians | Clinician-carer communication tool | Models of care | Implementation | Examines the impact of implementing a clinician-carer communication tool for hospitalised patients with dementia |  |  |  |
| Stevens (2012) | USA | Translation study, Quantitative  (before/after survey) | 1 hospital,  1 internal medicine primary care clinic, 164 ppts | PwD and their caregivers | Translation of the REACH II caregiver intervention | Knowledge transfer and dementia education | Implementation | Describes the creation and initial findings of a caregiver programme based on the REACH II intervention adapted for a healthcare setting using the RE-AIM framework |  |  |  |
| **Primary care (n=8)** | | | | | | | | |  |  |  |
| Bamford (2014) | UK | Ethnographic methods (interviews, observation) | 49 ppts | Various stakeholders inc. PwD, caregivers HCPs | Case management | Care practices | NA | To explore factors influencing the delivery of case management in practice using the Normalisation Process Theory framework |  |  |  |
| Cherry (2004) | USA | Quality improvement project,  Quantitative (before/after survey) | 112/126 surveys,  83 PwD and their caregivers | Physicians, social workers, | Practice guidelines for dementia diagnosis and management | Guideline-driven practices | Both | To improve dementia care quality in a Kaiser Permanente service area through dissemination and implementation of diagnostic and management practice guidelines |  |  |  |
| Lee (2013) | Canada | Quantitative  (before/after survey) | 22 clinics, 124 ppts | Various health professionals | Training programme for development of memory clinics | Services and infrastructure | Both | To describe a training program to build capacity within primary care settings for dementia assessment and management through the development of memory clinics |  |  |  |
| Lee (2014) | Canada | Qualitative  (interviews) | 40 ppts | Various health professionals | Development of interprofessional memory clinics | Services and infrastructure | NA | To describe factors affecting the development, implementation and sustainability of memory clinics |  |  |  |
| Mattiusi (2012) | Argentina | Qualitative (vignette, interviews, focus groups) | 52 ppts | Physicians | Advanced Directives | Care directives/frameworks | NA | To elicit physicians’ views about the discussion of Advanced Directives with PwD in the early stage |  |  |  |
| Murphy (2014) | Australia | Qualitative  (interviews) | 30 ppts | GPs | Guideline implementation for dementia diagnosis and management | Guideline-driven practices | NA | To explore and describe the barriers and facilitators to two guideline-recommended practices in dementia diagnosis and management using the Theoretical Domains Framework |  |  |  |
| Pimplott (2009a) | Canada | Qualitative  (focus groups) | 18 ppts | Family physicians | a) clinical practice guidelines | Guideline-driven practices | NA | To assess awareness, attitudes and use of clinical practice guidelines, and explore barriers and facilitators to implementation |  |  |  |
| Vollmar (2010) | Germany | Quantitative  (cluster RCT) | 30 clusters, 187 ppts | GPs | Dementia management knowledge | Knowledge transfer and dementia education | Dissemination | Compares knowledge acquisition about dementia management between a blended learning approach using online modules in addition to quality circles and quality circles alone |  |  |  |
| **Community care (n=16)** | | | | | | | | |  |  |  |
| Argyle (2015) | UK | Qualitative  (interviews) | 5 staff | Staff involved in project management | Person-centred care intervention | Models of care | NA | To examine the barriers and facilitators in the process of implementing a personalised musical intervention within a specialist dementia home care service |  |  |  |
| Boughtwood (2012) | Australia | Qualitative  (focus groups, interviews) | 181 ppts | Family carers, GPs, geriatricians, healthcare workers | Access to dementia information | Knowledge transfer and dementia education | Dissemination | Examines the perspectives of culturally and linguistically diverse family caregivers and other stakeholders on sources of information, issues of access and ways to improve information provision |  |  |  |
| Chee (2007) | USA | Quantitative  (prospective observational study) | 105 ppts | Caregivers | Predictors of adherence to caregiver intervention | Care practices | NA | Evaluates treatment implementation factors as predictors of caregiver adherence to skills training intervention guided by the Treatment Implementation framework and behaviour change models |  |  |  |
| Connell (2002) | USA | Mixed (before/after survey, focus groups) | 28 ppts | HCPs | Community outreach education | Knowledge transfer and dementia education | Dissemination | Describes the process of disseminating and evaluating the Dementia Education Train-the-Trainer programme in community rural areas |  |  |  |
| Gitlin (2010) | USA | Translation study,  Mixed (survey) | 64 ppts | Occupational therapists family caregivers | Translation of caregiver intervention into private homecare practice | Knowledge transfer and dementia education | Implementation | To evaluate the implementation and translation of a skill building programme for delivery by occupational therapists in private practice using the RE-AIM framework |  |  |  |
| Glasby (2003) | UK | Qualitative  (interviews) | 36 ppts | Social services managers, local specialists | Provision of information to carers | Knowledge transfer and dementia education | NA | To investigate barriers to the provision of information to carers of PwD and suggested solutions to overcome them |  |  |  |
| Lachenmayr (2000) | USA | Quantitative  (cross-sectional observation study) | 319 police departments | Police officers | Community partnership outreach programme for wandering behaviour in PwD | Knowledge transfer and dementia education | Both | To describe the design, implementation and impact evaluation of a health education partnership with police officers to increase the safety of people with AD who wander using principles from the Diffusion of Innovation and Social Learning theories |  |  |  |
| Manthorpe (2013) | UK | Qualitative  (focus groups, interviews) | 272 practitioners | Social care staff | Mental Capacity Act | Care directives/frameworks | NA | To explore the experiences and practice challenges of social care staff in implementing the MCA for PwD |  |  |  |
| McCurry (2003) | USA | Quantitative  (RCT) | 22 PwD and their caregivers | Caregivers | Sleep hygiene recommendations | Care practices | Implementation | To examine the feasibility of training caregivers to implement sleep hygiene changes with PwD |  |  |  |
| Meiland (2005) | Netherlands | Qualitative  (interviews, logs, project reports) | 13 meeting centres,  23 ppts | Various staff, members of organisations and financial experts | Implementation of meeting centres | Services and infrastructure | NA | To investigate facilitating and impeding factors in the adaptive implementation of the meeting centres programme to support PwD and their carers presented according to the different levels of the implementation process |  |  |  |
| Samia (2014) | USA | Translation study,  Quasi experimental  (before/after survey, focus groups) | 16 counties, 37 trainers | Trainee family caregivers | Translation of caregiver training programme | Knowledge transfer and dementia education | Implementation | To evaluate the implementation and translation of a psychoeducation programme for family caregivers across Maine counties using the RE-AIM framework |  |  |  |
| Samsi (2012) | UK | Qualitative  (interviews) | 15 ppts | Admiral nurses | Mental Capacity Act | Care directives/frameworks | NA | To explore experiences, expectations and challenges of admiral nurses providing information about the Mental Capacity Act to PwD and their carers |  |  |  |
| Van Haeften (2015b) | Netherlands | Process evaluation,  Qualitative (interviews) | 6 centres, 40 stakeholders | Managers, project leaders, HCPs | Transformation of day care centres | Services and infrastructure | Implementation | To determine facilitating and impeding factors of the transition from nursing home-based day care to community-based day care centres |  |  |  |
| Van Mierlo (2014) | Netherlands | Process evaluation, Qualitative  (interviews) | 22 ppts | Various stakeholders | Delivery of case management models | Care practices | NA | To explore barriers and facilitators to the delivery of community-based personalised dementia care of two different case management models |  |  |  |
| Van’t Leven (2012) | Netherlands | Qualitative  (focus groups) | 31 ppts | Occupational therapists, physicians, managers | Occupational therapy guidelines implementation | Guideline-driven practices | NA | To identify the barriers and facilitators to the implementation of the proven Community Occupational Therapy in Dementia (COTiD) guideline for PwD and their carers |  |  |  |
| While (2010) | Australia | Mixed  (before/after survey, focus groups) | 66/47,  35 ppts | District nurses | Dementia education programme | Knowledge transfer and dementia education | Dissemination | Describes the development and evaluation of a dementia education programme to support new clinical practice |  |  |  |
| **Multiple settings (n=13)** | | | | | | | | |  |  | **Multiple settings (n=13)** |
| Clarke (2014) | UK | Mixed  (surveys, interviews) | 40 demonstration sites | Various staff, stakeholders,  caregivers | National evaluation of new demonstration sites | Services and infrastructure | NA | To report organisational mechanisms and challenges in the implementation of the Dementia Advisers and Peer Support Networks services in 40 demonstrator sites established under the National Dementia Strategy for England |  |  |  |
| Döpp (2013a) | Netherlands | Quantitative  (cluster RCT) | 45 clusters, 222 ppts | Physicians, managers, occupational therapists | Multifaceted implementation strategy | Knowledge transfer and dementia education | Implementation | To evaluate the effectiveness of a multifaceted implementation strategy on physicians’ referral rate to and knowledge on the community occupational therapy programme |  |  |  |
| Döpp (2013b) | Netherlands | Process evaluation,  Mixed (questionnaire, interviews, focus groups) | unclear | Physicians, managers, occupational therapists | Multifaceted implementation strategy | Knowledge transfer and dementia education | NA | To identify factors influencing the effectiveness of the multifaceted implementation strategy of the community occupational therapy programme |  |  |  |
| Döpp (2015) | Netherlands | Quantitative  (cluster RCT) | 94 ppts | Occupational therapists | Multifaceted implementation strategy | Knowledge transfer and dementia education | Implementation | To evaluate the effectiveness of a multifaceted implementation strategy on occupational therapists’ adherence to the community occupational therapy programme |  |  |  |
| Fortune (2015) | Canada | Action research, Qualitative  (interviews) | 2 care settings,  25 ppts | Staff, researchers, family members, volunteers | Culture change of dementia care | Models of care | NA | To examine the complexities and challenges of a participatory action research project aiming at changing the culture of dementia care in 2diverse care settings to reflect a relationship-centred approach |  |  |  |
| Kumpers (2006) | Netherlands/UK | Qualitative  (case studies, interviews) | 4 case-studies, 100 ppts | HCPs, managers, carers | Knowledge transfer between services | Knowledge transfer and dementia education | Dissemination | To identify factors associated with success and failure of knowledge transfer between specialist and generic services using an existing performance model and a cross-national sample |  |  |  |
| Lee (2015) | UK | Qualitative  (interviews) | 30 ppts | Experts in dementia care with various roles | Palliative and dementia care | Models of care | NA | To determine expert views on key factors influencing good practice in end of life care for PwD |  |  |  |
| Lewis (2005) | Canada | Quantitative  (survey) | 14 organisations, 34 staff | Various staff in specialised geriatric services | Guidelines for non -pharmaco logical dementia management | Guideline-driven practices | Both | To describe approaches used to disseminate and implement clinical practice guidelines for dementia management to staff working in specialised geriatric services, and barriers and facilitators to adherence |  |  |  |
| Paone (2014) | USA | Process evaluation,  Mixed (surveys, interviews) | 14 program sites, 18 consultants | Trainee consultants, spousal caregivers | Translation of caregiver intervention | Knowledge transfer and dementia education | Implementation | To evaluate the implementation and translation challenges of a caregiver intervention designed to support spousal caregivers of persons with AD using the RE-AIM framework |  |  |  |
| Robinson (2013) | UK | Qualitative  (focus groups, interviews) | 95 ppts | HCPs | Advance care planning | Care directives/frameworks | NA | To explore professionals views and experiences on the implementation of advanced care planning |  |  |  |
| Rolnick (2013) | USA | Qualitative  (interviews) | 11 ppts | Health care providers | Communicating incontinence | Care practices | NA | To examine healthcare providers’ perspectives regarding improving communication with patients and their caregivers about incontinence and skin damage in PwD |  |  |  |
| Vasse (2011) | Europe | Qualitative  (focus groups) | 9 countries, 27 ppts | Dementia care professionals and researchers | International QIs for psychosocial care | Models of care | NA | To explore potential barriers and facilitators to the implementation of a set of Quality Indicators of evidence-based psychosocial dementia care across various European countries and to compose an implementation strategy |  |  |  |
| Yusoff (2013) | Malaysia | Quantitative  (before/after study) | 59 ppts | HCPs | Clinical practice guidelines for dementia management | Guideline-driven practices | Both | To evaluate change in knowledge and understanding of dementia following training based on the Malaysian clinical practice guidelines and evaluate the effectiveness of the training sessions according to a clinical audit indicator |  |  |  |
| BPSD, Behavioural and psychological symptoms of dementia; CHs, care homes; GPs, general practitioners; HCPs, health care professionals; LTC, long-term care; NA, not applicable; NHs, nursing homes; ppts, participants; PwD, people with dementia; RCT, randomised controlled trial | | | | | | | | |  |  |  |
