# Supplementary material for: Dissemination and implementation research in dementia care: a systematic scoping review and evidence map
Source: BMC Geriatr. 2017 Jul 14;17:147. doi: 10.1186/s12877-017-0528-y (PMC5513053; doi:10.1186/s12877-017-0528-y)
Supplement: Supplementary file 3 — Implementation strategies identified across studies (n = 52) coded based on the ERIC compilation. (DOCX 19 kb) [file 12877_2017_528_MOESM3_ESM.docx]

| **Table S2. Implementation strategies identified across studies (n=52) based on the ERIC compilation** | |
| --- | --- |
| **Implementation strategies** | **No. of times identified** |
| 1.Use evaluative and iterative strategies | 24 |
| 4 Assess for readiness and identify barriers and facilitators  5 Audit and provide feedback  56 Purposefully re-examine the implementation  26 Develop and implement tools for quality monitoring  27 Develop and organise quality monitoring systems  23 Develop a formal implementation blueprint  18 Conduct local needs assessment  61 Stage implementation scale up  46 Obtain and use patients/consumers and family feedback  14 Conduct cyclical small tests of change | 6  6  2  5  1  1  7  3  5  3 |
| 2.Provide interactive assistance | 11 |
| 33 Facilitation  54 Provide local technical assistance  53 Provide clinical supervision  8 Centralise technical assistance | 7  2  4  0 |
| 3.Adapt and tailor to context | 19 |
| 63 Tailor strategies  51 Promote adaptability  67 Use data experts  68 Use data warehousing techniques | 8  11  0  0 |
| 4.Develop stakeholder interrelationships | 36 |
| 35 Identify and prepare champions  48 Organize clinician implementation team meetings  57 Recruit, designate, and train for leadership  38 Inform local opinion leaders  6 Build a coalition  47 Obtain formal commitments  36 Identify early adopters  17 Conduct local consensus discussions  7 Capture and share local knowledge  64 Use advisory boards and workgroups  65 Use an implementation advisor  45 Model and simulate change  72 Visit other sites  40 Involve executive boards  25 Develop an implementation glossary  24 Develop academic partnerships  52 Promote network weaving | 7  6  6  2  4  2  0  5  2  13  1  0  1  3  0  0  2 |
| 5.Train and educate stakeholders | 49 |
| 19 Conduct ongoing training  55 Provide ongoing consultation  29 Develop educational materials  43 Make training dynamic  31 Distribute educational materials  71 Use train-the-trainer strategies  15 Conduct educational meetings  16 Conduct educational outreach visits  20 Create a learning collaborative  60 Shadow other experts  73 Work with educational institutions | 6  16  17  19  34  9  38  9  10  4  1 |
| 6.Support clinicians | 14 |
| 32 Facilitate relay of clinical data to providers  58 Remind clinicians  30 Develop resource sharing agreements  59 Revise professional roles  21 Create new clinical teams | 0  5  5  3  1 |
| 7.Engage consumers | 10 |
| 41 Involve patients/consumers and family members  39 Intervene with patients/consumers to enhance uptake and adherence  50 Prepare patients/consumers to be active participants  37 Increase demand  69 Use mass media | 7  1  0  1  2 |
| 8.Utilise financial strategies | 8 |
| 34 Fund and contract for the clinical innovation  1 Access new funding  49 Place innovation on fee for service lists/formularies  2 Alter incentive/allowance structures  42 Make billing easier  3 Alter patient/consumer fees  70 Use other payment schemes  28 Develop disincentives  66 Use capitated payments | 1  6  1  0  0  0  0  0  0 |
| 9.Change infrastructure | 15 |
| 44 Mandate change  12 Change record systems  11 Change physical structure and equipment  22 Create or change credentialing and/or licensure standards  13 Change service sites  9 Change accreditation or membership requirements  62 Start a dissemination organization  10 Change liability laws | 3  8  5  3  1  0  0  0 |
