# Supplementary material for: Dissemination and implementation research in dementia care: a systematic scoping review and evidence map
Source: BMC Geriatr. 2017 Jul 14;17:147. doi: 10.1186/s12877-017-0528-y (PMC5513053; doi:10.1186/s12877-017-0528-y)
Supplement: Supplementary file 4 — Summary of implementation strategies and outcomes across studies categorised by care setting. (DOCX 24 kb) [file 12877_2017_528_MOESM4_ESM.docx]

| **Table S3. Summary of implementation strategies and outcomes across studies by care setting** | | | | | |
| --- | --- | --- | --- | --- | --- |
| **First author**  **(year)** | **Broad category of study focus** | **Clusters (#1-9)** | **Implementation strategies**  **(#1-73)** | **Type of outcome** | **Barriers and/or facilitators to implementation** |
| **Residential long-term care (n=46)** | | | | | |
| Andrews (2009) | Guideline-driven practices | 1,3,4,5,7 | 14,18,20,29,31,41,46,48,63 | Professional: Performance  Family: Perceptions | B: Organisational |
| Aveyard (2006) | Models of care | - | - | Barriers to change | B: Organisational, Individual, Financial |
| Brooker (2014) | Behaviour management | 2,4,5 | 15,20,53,57,73 | Professional: Treatment, Knowledge, Attitudes | B&F: Organisational, Professional, Individual |
| Burgio (2002) | Behaviour management | 2,5 | 15,31,43, 53,54,55 | Professional: Compliance, Performance, Knowledge  PwD: Behaviour | **-** |
| Calleson (2006) | Care practices | 5,7,8,9 | 1,22,29,31,43,69 | Professional: Performance, Knowledge | **-** |
| Chenoweth (2015) | Models of care | - | **-** | Patient: Behaviour  Family: Perceptions | B&F: Organisational, Individual |
| Cooke (2014) | Models of care | 4,5 | 15,29,31,55,57,71 | Professional: Perceptions | B&F: Organisational, Professional |
| Dickson (2015) | Care practices | 1,5 | 14,15,18,43 | Professional: Treatment, Knowledge | - |
| Fallon (2006) | Care practices | 1,3,4,5 | 4,14,15,23,26,31,43 ,51,64,71 | Professional: Compliance, Knowledge, Perceptions | B: Organisational, Professional |
| Finucane (2013) | Models of care | 1,4,5 | 15,26,35,55,60 | Professional: Compliance  PwD: Deaths | B: Organisational |
| Fleming (2012) | Services and infrastructure | - | **-** | Barriers to change | B: Organisational, Individual, Financial |
| Gnaedinger (2003) | Models of care | - | **-** | Barriers & facilitators to change | B&F: Organisational, Professional, Individual |
| Hoffman (1998) | Knowledge transfer and dementia education | - | **-** | Barriers to change | B: Organisational, Professional |
| Holle (2015) | Behaviour management | - | **-** | Barriers to change | B: Organisational, Professional |
| Hynes (2014) | Behaviour management | 1,3,4,5,6,9 | 4,5,12,15,17, 30,31,63,64 | Professional: Assessment, Compliance | B: Professional, Individual |
| Janzen (2013) | Behaviour management | - | - | Barriers & facilitators to change | B&F: Organisational, Individual |
| Kolanowski (2015) | Models of care | - | - | Barriers to change | B: Organisational, Professional |
| Kovach (2008) | Care practices | 1,4,5 | 4,15,29,31 ,48,55,61 | Professional: Assessment, Treatment  PwD: Behaviour | B&F: Organisational, Professional, Individual |
| Lawrence (2015) | Behaviour management | - | - | Barriers & facilitators to change | B&F: Organisational, Individual |
| Mann (2013) | Care directives/frameworks | - | - | Barriers & facilitators to change | B&F: Professional, Individual |
| Mellor (2015) | Behaviour management | 2,5 | 15,33,55 | Professional: Compliance, Perceptions  PwD: Behaviour | - |
| Monette (2008) | Behaviour management | 1,4,5,6 | 15,26,31,35,58 | Professional: Treatment, Performance  PwD: Behaviour | - |
| Morgan (2005) | Services and infrastructure | - | - | Barriers & facilitators to change | B&F: Organisational, Professional, Environmental |
| Moyle (2013) | Models of care | 2,4,5,7 | 15,16,41,55,57,71 | Professional: Perceptions | B&F: Organisational, Individual |
| Munir (2007) | Care practices | 1,4,6 | 18, 56,58,64 | Professional: Treatment | **-** |
| Murray (2011) | Behaviour management | 1,3,4,5,9 | 4,5,11,15 ,27,29,,43,48,63 | Professional: Assessment, Compliance | **-** |
| Oye (2015) | Behaviour management | 2,5 | 15,33,55 | Professional: Attitudes | B&F: Organisational, Individual |
| Ranasinghe  (2013) | Care practices | 1,3,4,5,7,9 | 4,5,12,15,31,41,63,64 | Professional: Assessment, Compliance | F: Professional |
| Rapp (2013) | Guideline-driven practices | 3,5 | 15,29,63 | Professional: Treatment  PwD: Behaviour | - |
| Roberts  (2015) | Models of care | 1,4,5,6,7,9 | 11,15,21,35,41,46,55,57,64 | Professional: Treatment, Knowledge  PwD: Behaviour | - |
| Rokstad (2015) | Models of care | - | - | Facilitator of change | F: Organisational |
| Rooney (2014) | Guideline-driven practices | 1,3,4,5,8 | 1,4,5,15,31,63,64 | Professional: Assessment, Compliance, Knowledge | - |
| Sacoco (2014) | Care practices | 1,4,5,9 | 12,15,18,19,26,29,31,40,43, 61 | Professional: Assessment, Compliance | F: Organisational |
| Sidani (2009) | Models of care | 5 | 15,31,43 | Professional: Compliance | - |
| Simpson (2007) | Care practices | - | - | Barriers & facilitators to change | B&F: Organisational, Professional, Individual |
| Slaughter (2013) | Care practices | 1,5,6,9 | 5,12,16,31,58 | PwD: Behaviour | - |
| Sung (2008) | Behaviour management | 4,5,6 | 17,31,38,43,58 | Professional: Compliance, Knowledge | - |
| Teri (2009) | Knowledge transfer and dementia education | 3.5 | 15,20,31,43,51,55,71 | Barriers to change | B: Organisational, Professional, Individual |
| Timmins (2008) | Behaviour management | 1,3,4,5,9 | 11,12,26, 29,31,44,63,64 | Professional: Assessment, Compliance, Knowledge | B: Organisational, Individual |
| Tjia (2015) | Guideline-driven practices | 1,4,5 | 5,15,16,17,18,29,31,47 | Professional: Compliance, Treatment | - |
| Van der Kooij (2013) | Models of care | 2,4,5 | 15,53,57 | Professional: Performance | - |
| Van Haeften  (2015a) | Models of care | 4,5,6 | 7,15,19,20,30,55 | Barriers & facilitators to change | B&F: Organisational, Individual |
| Verkaik (2011) | Guideline-driven practices | 3,4,5 | 15,31,33,43,48,55,71 | Professional: Compliance | B&F: Organisational, Individual, Patient-specific |
| Vida (2012) | Behaviour management | 1,5 | 15,31,56 | Professional: Treatment  PwD; Behaviour | - |
| Vikstrom (2015) | Guideline-driven practices | 1,2,3,4,5 | 7,15,17,19,20,31,33,43,46,48,51 | Professional: Perceptions | B: Organisational |
| Zwijsen (2014) | Behaviour management | - | - | Barriers & facilitators to change | B&F: Organisational, Individual |
| **Hospitals (n=5)** | | | | | |
| Atkinson (2012) | Services and infrastructure | 1,3,4,6,9 | 11,12,18,44,51,64 | Professional: Compliance | - |
| Banks (2014) | Knowledge transfer and dementia education | 4,5 | 15,20,31,35,43,72 | Professional: Attitudes, Perceptions | - |
| Henderson (2006) | Knowledge transfer and dementia education | 2,4,5,9 | 11,16,17,31,33,35,60 | Professional: Attitudes | - |
| Luxford (2015) | Models of care | 3,4,5,7 | 15,16,31,35, 40,41,48,51 | Professional: Compliance, Treatment, Perceptions  PwD: Physical health | B&F: Organisational, Professional, Individual |
| Stevens (2012) | Knowledge transfer and dementia education | 1,2,3,4,5,9 | 6,12,16,19,29,33,51,58,61 | Professional: Compliance  Family: Behaviour, Compliance | - |
| **Primary care (n=8)** | | | | | |
| Bamford (2014) | Care practices | - | - | Barriers to change | B: Organisational, Individual |
| Cherry (2004) | Guideline-driven practices | 4,5,6,7 | 19,30,31,41,43,59,64, | Professional: Assessment, Compliance | - |
| Lee (2013) | Services and infrastructure | 3,4,5,8,9 | 1,15,20,22,31,43,51,52,55,60 | Professional: Compliance, Knowledge,Perceptions | - |
| Lee (2014) | Services and infrastrucutre | - | - | Barriers & facilitators to change | B&F: Organisational, Professional |
| Mattiusi (2012) | Care directives/frameworks | - | - | Barriers to change | B: Organisational, Individual, dementia-specific |
| Murphy (2014) | Guideline-driven practices | - | - | Barriers & facilitators to change | B&F: Organisational, Professional |
| Pimplott (2009a) | Guideline-driven practices | - | - | Barriers to change | B:Professional, Individual |
| Vollmar (2010) | Knowledge transfer and dementia education | 5,6 | 15,30,31,43 | Professional: Knowledge | - |
| **Community care (n=16)** | | | | | |
| Argyle (2015) | Models of care | - | - | Barriers & facilitators to change | B&F: Organisational, Professional |
| Boughtwood (2012) | Knowledge transfer and dementia education | - | - | Barriers to change | B: Organisational, Individual |
| Chee (2007) | Care practices | - | - | Barriers & facilitators to change | B&F: Organisational, Professional |
| Connell (2002) | Knowledge transfer and dementia education | 4,5 | 15,20,29,31,43,52,64,71 | Professional: Knowledge | - |
| Gitlin (2010) | Knowledge transfer and dementia education | 3,5,8 | 15,31,43,49,51,55 | Professional: Compliance, Performance, Perceptions  Family: Knowledge, Perceptions | B: Organisational |
| Glasby (2003) | Knowledge transfer and dementia education | - | - |  | B&F: Organisational, Professional, Individual |
| Lachenmayr (2000) | Knowledge transfer and dementia education | 4,5,6,7,9 | 6,15,29,30,31,38,40,43,44,65,69 | Professional: Compliance, Performance | - |
| Manthorpe (2013) | Care directives/frameworks | - | - | Barriers to change | B: Organisational |
| McCurry (2003) | Care practices | 2,3,5,7 | 16,31,33,39,41,51,55 | Family: Compliance | - |
| Meiland (2005) | Services and infrastructure | - | - | Barriers & facilitators to change | B&F: Organisational, Professional, Individual |
| Samia (2014) | Knowledge transfer and dementia education | 4,5,8 | 1,6,15,29,60,64,71 | Professional: Compliance, Performance, Perceptions | B&F: Organisational |
| Samsi (2012) | Care directives/frameworks | - | - | Barriers to change | B:Professional |
| Van Haeften (2015b) | Services and infrastructure | 3,4,5,9 | 6,13,15,47,51,55,64 | Barriers & facilitators to change | B&F: Organisational, Professional, Individual |
| Van Mierlo (2014) | Care practices | - | - | Barriers to change | B: Organisational |
| Van’t Leven (2012) | Guideline-driven practices | - | - | Barriers & facilitators to change | B&F: Organisational, Professional |
| While (2010) | Knowledge transfer and dementia education | 1,2,3,4,5,8 | 1,15,16,29,46,51,53, 71 | Professional: Knowledge, Perceptions | F: Organisational |
| **Multiple settings (n=13)** | | | | | |
| Clarke (2014) | Services and infrastrucuture | - | - | Barriers & facilitators to change | B&F: Organisational |
| Döpp (2013a) | Knowledge transfer and dementia education | 3,5,6,8,9 | 1,12,15,16,20,22,31,58,63 | Professional: Compliance, Knowledge | - |
| Döpp (2013b) | Knowledge transfer and dementia education | - | - | Barriers & facilitators to change | B&F: Organisational, Individual |
| Döpp (2015) | Knowledge transfer and dementia education | 3,5,6,8,9 | 1,12,15,16,20,22,31,58,63 | Professional: Compliance | - |
| Fortune (2015) | Models of care | - | - | Barriers to change | B: Organisational, Individual |
| Kumpers (2006) | Knowledge transfer and dementia education | - | - | Barriers & facilitators to change | B&F: Organisational, Professional |
| Lee (2015) | Models of care | - | - | Barriers & facilitators to change | B&F: Organisational |
| Lewis (2005) | Guideline-driven practices | 1,4,5 | 18,20,29,31,35,64 | Professional: Compliance, Knowledge | B&F: Organisational, Professional |
| Paone (2014) | Knowledge transfer and dementia education | 1,2,5,6,7,8 | 19,29,31,34,37,46,54,55,59 | Professional: Compliance, Performance  Family: Perceptions | B&F: Organisational, Professional |
| Robinson (2013) | Care directives/frameworks | - | - | Barriers to change | B: Organisational, Professional, Legal |
| Rolnick (2013) | Care practices | - | - | Barriers & facilitators to change | B&F: Organisational, Professional, Individual |
| Vasse (2011) | Models of care | - | - | Barriers & facilitators to change | B&F: Organisational, Professional, Individual |
| Yusoff (2013) | Guideline-driven practices | 4,5 | 15,29,31,43,57,71 | Professional: Compliance, Knowledge | - |
| B; barrier(s); F, facilitator(s); B&F, barriers and facilitators; BPSD, Behavioural and psychological symptoms of dementia; PwD, people with dementia; QIs, quality indicators | | | | | |
