# Supplementary material for: Dissemination and implementation research in dementia care: a systematic scoping review and evidence map
Source: BMC Geriatr. 2017 Jul 14;17:147. doi: 10.1186/s12877-017-0528-y (PMC5513053; doi:10.1186/s12877-017-0528-y)
Supplement: Supplementary file 5 — List of studies included in the systematic scoping review (n = 88). (PDF 174 kb) [file 12877_2017_528_MOESM5_ESM.pdf]

Appendix B

List of studies included in the scoping review (n=88):

1. Andrews, S., F. McInerney, and A. Robinson, *Realizing a palliative approach in dementia care: Strategies to facilitate aged care staff engagement in evidence-ba ed practice.* International Psychogeriatrics, 2009. **21**(SUPPL. 1): p. S64-S68.

2. Argyle, E. and T. Kelly, *Implementing person centred dementia care: a musical intervention.* Working with Older People, 2015. **19**(2): p. 77-84.

3. Atkinson, P. and E.B. Mukaetova-Ladinska, *Nurse-led liaison mental health service for older adults: service development using lean thinking methodology.* Journal of Psychosomatic Research, 2012. **72**(4): p. 328-31.

4. Aveyard, B. and S. Davies, *Moving forward together: evaluation of an action group involving staff and relatives within a nursing home for older people with dementia.* International Journal of Older People Nursing, 2006. **1**(2): p. 95-104.

5. Bamford, C., M. Poole, and K. Brittain, *Understanding the challenges to implementing case management for people with dementia in primary care in England : a qualitative study using Normalization Process Theory.* BMC Health Services, 2014. **14**(549).

6. Boughtwood, D., et al., *Dementia information for culturally and linguistically diverse communities: Sources, access and considerations for effective practice.* Australian Journal of Primary Health, 2012. **18**(3): p. 190-196.

7. Brooker, D. and et al., *"FITS into practice": developing dementia specialist care homes: summary report.* 2014: p. 27.

8. Calleson, D.C., P.D. Sloane, and L.W. Cohen, *Effectiveness of mailing "bathing without a battle" to all US nursing homes.* Gerontology and Geriatrics Education, 2006. **27**(1): p. 67-79.

9. Chee, Y.K., et al., *Predictors of adherence to a skill-building intervention in dementia caregivers.* Journals of Gerontology Series A-Biological Sciences & Medical Sciences, 2007. **62**(6): p. 673-8.

10. Chenoweth, L., et al., *Percen trial participant perspectives on the implementation and outcomes of person-centered dementia care and environments.* International Psychogeriatrics, 2015: p. No Pagination Specified.

11. Cherry, D.L., et al., *Interventions to improve quality of care: The Kaiser Permanente-Alzheimer's Association dementia care project.* American Journal of Managed Care, 2004. **10**(8): p. 553-560.

12. Clarke, C., S. Keyes, and H. Wilkinson, *Organisational space for partnership and sustainability : lessons from the implementation of the National Dementia Strategy for England.* Health and Social Care in the Community, 2014. **22**(6): p. 634-645.

13. Connell, C.M., et al., *Providing dementia outreach education to rural communities: Lessons learned from a Train-the-Trainer Program.* Journal of Applied Gerontology, 2002. **21**(3): p. 294-313.

14. Cooke, M., et al., *Evaluation of an education intervention to implement a capability model of dementia care.* Dementia-International Journal of Social Research and Practice, 2014. **13**(5): p. 613-625.

15. Dickson, T.A., *Implementing a pain assessment program among older adults with cognitive impairment.* Dissertation Abstracts International: Section B: The Sciences and Engineering, 2015. **75**(9-B(E)): p. No Pagination Specified.

16. Dopp, C.M., et al., *Determinants for the effectiveness of implementing an occupational therapy intervention in routine dementia care.* Implementation science: 2013. **8**: p. 131.

17. Dopp, C.M., et al., *Effectiveness of a multifaceted implementation strategy on physicians' referral behavior to an evidence-based psychosocial intervention in dementia: a cluster randomized controlled trial.* BMC family practice, 2013. **14**: p. 70.

18. Dopp, C.M., et al., *Effectiveness of a training package for implementing a community-based occupational therapy program in dementia: a cluster randomized controlled trial.* Clinical Rehabilitation, 2015. **29**(10): p. 974-86.

19. Fallon, T., et al., *Implementation of oral health recommendations into two residential aged care facilities in a regional Australian city.* International Journal of Evidence-Based Healthcare, 2006. **4**(3): p. 162-79.

20. Finucane, A.M., et al., *Improving end-of-life care in nursing homes: Implementation and evaluation of an intervention to sustain quality of care.* Palliative Medicine, 2013. **27**(8): p. 772-778.

21. Fleming, R., R. Fay, and A. Robinson, *Evidence-based facilities design in health care: A study of aged care facilities in Australia.* Health Services Management Research, 2012. **25**(3): p. 121-128.

22. Fortune, D., et al., *"It was like reading a detective novel": Using PAR to work together for culture change.* Journal of Aging Studies, 2015. **34**: p. 38-47.

23. Gitlin, L.N., M. Jacobs, and T.V. Earland, *Translation of a dementia caregiver intervention for delivery in homecare as a reimbursable Medicare service: outcomes and lessons learned.* The Gerontologist, 2010. **50**(6): p. 847-854.

24. Glasby, J. and L. Kilbride, *Who knows?* Practice, 2003. **15**(4).

25. Henderson, A., et al., *The motivation of health professionals to explore research evidence in their practice: An intervention study.* Journal of Clinical Nursing, 2006. **15**(12): p. 1559-1564.

26. Hoffman, S.B. and M. Kaplan, *Problems encountered in the implementation of dementia care programs.* American Journal of Alzheimer's Disease, 1998. **13**(4): p. 197-202.

27. Holle, D., et al., *Experiences of nursing staff using dementia-specific case conferences in nursing homes.* American Journal of Alzheimer's Disease and other Dementias, 2015. **30**(3): p. 228-237.

28. Hynes, A., et al., *Assessment and Non-Pharmacological management among adults with a dementia diagnosis in a residential care setting: A best practice implementation project.* JBI Database of Systematic Reviews and Implementation Reports, 2014. **12**(9): p. 489-501.

29. Janzen, S., et al., *Managing agitation using nonpharmacological interventions for seniors with dementia.* American Journal of Alzheimer's Disease and other Dementias, 2013. **28**(5): p. 524-532.

30. Kolanowski, A., et al., *"Wish we would have known that!" Communication Breakdown Impedes Person-Centered Care.* Gerontologist, 2015. **55 Suppl 1**: p. S50-60.

31. Kovach, C.R., et al., *Using principles of diffusion of innovation to improve nursing home care.* Journal of Nursing Care Quality, 2008. **23**(2): p. 132-9.

32. Kumpers, S., *The importance of knowledge transfer between specialist and generic services in improving health care : a cross-national study of dementia care in England and the Netherlands.* International Journal of Health Planning and Management, 2006. **21**(2): p. 151-167.

33. Lachenmayr, S., K.D. Goldman, and F.S. Brand, *Safe return: a community-based initiative between police officers and the Alzheimer's Association to increase the safety of people with Alzheimer's disease.* Health Promotion Practice, 2000. **1**(3): p. 268-278.

34. Lawrence, V., et al., *Helping staff to implement psychosocial interventions in care homes: Augmenting existing practices and meeting needs for support.* International Journal of Geriatric Psychiatry, 2015: p. No Pagination Specified.

35. Lee, L., L.M. Hillier, and W.W. Weston, *Ensuring the success of interprofessional teams: key lessons learned in memory clinics.* Canadian journal on aging = La revue canadienne du vieillissement, 2014. **33**(1): p. 49-59.

36. Lee, L., W.W. Weston, and L.M. Hillier, *Developing memory clinics in primary care: An evidence-based interprofessional program of continuing professional development.* Journal of Continuing Education in the Health Professions, 2013. **33**(1): p. 24-32.

37. Lee, R.P., et al., *Expert views on the factors enabling good end of life care for people with dementia: A qualitative study.* BMC Palliative Care, 2015. **14**(1).

38. Lewis, D.L., et al., *Translating evidence into practice: the case of dementia guidelines in specialized geriatric services.* Canadian Journal on Aging, 2005. **24**(3): p. 251-60.

39. Luxford, K., A. Axam, and F. Hasnip, *Improving clinician–carer communication for safer hospital care : a study of the 'TOP 5' strategy in patients with dementia.* International Journal for Quality in Health Care, 2015. **27**(3): p. 175-182.

40. Mann, E., et al., *Do-Not-Hospitalize Orders for Individuals with Advanced Dementia: Healthcare Proxies' Perspectives.* Journal of the American Geriatrics Society, 2013. **61**(9): p. 1568-1573.

41. Mattiussi, M., et al., *Physicians' views and perspectives on advanced directives in patients with incipient dementia.* Medicina (Argentina), 2012. **72**(4): p. 305-314.

42. McCurry, S.M., et al., *Training caregivers to change the sleep hygiene practices of patients with dementia: The NITE-AD project.* Journal of the American Geriatrics Society, 2003. **51**(10): p. 1455-1460.

43. Meiland, F.J.M., et al., *Facilitators and barriers in the implementation of the meeting centres model for people with dementia and their carers.* Health Policy, 2005. **71**(2): p. 243-253.

44. Monette, J., et al., *Effect of an interdisciplinary educational program on antipsychotic prescribing among nursing home residents with dementia.* International Journal of Geriatric Psychiatry, 2008. **23**(6): p. 574-579.

45. Morgan, D.G., et al., *Creating and sustaining dementia special care units in rural nursing homes: the critical role of nursing leadership.* Canadian journal of nursing leadership, 2005. **18**(2): p. 74-99.

46. Moyle, W., et al., *Promoting value in dementia care: staff, resident and family experience of the capabilities model of dementia care.* Aging & mental health, 2013. **17**(5): p. 587-594.

47. Munir, J., R.J. Wright, and D.B. Carr, *A Quality Improvement Study on Calcium and Vitamin D Supplementation in Long-term Care.* Journal of the American Medical Directors Association, 2007. **8**(3 SUPPL. 2): p. e19-e23.

48. Murphy, K., et al., *Understanding diagnosis and management of dementia and guideline implementation in general practice: a qualitative study using the theoretical domains framework.* Implementation science : IS, 2014. **9**: p. 31.

49. Murray, R., *Implementing restraint minimization practices in a 30-bed high-care dementia unit: a best practice implementation project.* Pacesetters, 2011. **8**(3): p. 25-33.

50. Gnaedinger, N., *Changes in long-term care for elderly people with dementia: a report from the front lines in British Columbia, Canada.* Journal of Social Work in Long-Term Care, 2003. **2**(3/4): p. 355-371.

51. Paone, D., *Using RE-AIM to Evaluate Implementation of an Evidence-Based Program: A Case Example From Minnesota.* Journal of Gerontological Social Work, 2014. **57**(6/7): p. 602-625.

52. Pimlott, N.J., et al., *Family physicians and dementia in Canada: Part 1. Clinical practice guidelines: awareness, attitudes, and opinions.* Canadian Family Physician, 2009. **55**(5): p. 506-7.e1-5.

53. Ranasinghe, D., L. Bates, and P. Mynes, *Clinical risk assessment and management strategies for residents with severe dementia in a residential care setting: A best practice implementation project.* JBI Library of Systematic Reviews, 2013. **11**(7): p. 422-433.

54. Rapp, M.A., et al. *Agitation in nursing home residents with dementia (VIDEANT trial): effects of a cluster-randomized, controlled, guideline implementation trial*. Journal of the American Medical Directors Association, 2013. **14**, 690-5.

55. Robinson, A., et al., *Information pathways into dementia care services: Family carers have their say.* Dementia: The International Journal of Social Research and Practice, 2009. **8**(1): p. 17-37.

56. Rokstad, A.M., et al., *The role of leadership in the implementation of person-centred care using Dementia Care Mapping: a study in three nursing homes.* Journal of Nursing Management, 2015. **23**(1): p. 15-26.

57. Rolnick, S., et al., *Healthcare providers' perspectives on communicating incontinence and skin damage information with patients with dementia and their family caregivers: a descriptive study.* Ostomy/wound management, 2013. **59**(4): p. 62-67.

58. Rooney, C., *Management of physical aggression in the dementia resident in an 18 bed secure dementia unit: A best practice implementation project.* JBI Database of Systematic Reviews and Implementation Reports, 2014. **12**(8): p. 394-410.

59. Sacoco, C. and S. Ishikawa, *Evidence-Based Practice for Pain Identification in Cognitively Impaired Nursing Home Residents.* Nursing Clinics of North America, 2014. **49**(3): p. 345-356.

60. Samia, L.W., et al., *The Maine Savvy Caregiver Project: translating an evidence-based dementia family caregiver program within the RE-AIM Framework.* Journal of Gerontological Social Work, 2014. **57**(6-7): p. 640-61.

61. Samsi, K., et al., *Challenges and expectations of the Mental Capacity Act 2005: An interview-based study of community-based specialist nurses working in dementia care.* Journal of Clinical Nursing, 2012. **21**(11-12): p. 1697-1705.

62. Sidani, S., C. Leclerc, and D. Streiner, *Implementation of the abilities-focused approach to morning care of people with dementia by nursing staff.* International Journal of Older People Nursing, 2009. **4**(1): p. 48-56.

63. Simpson, M.R., P. Stevens, and C.R. Kovach, *Nurses' experience with the clinical application of a research-based nursing protocol in a long-term care setting.* Journal of Clinical Nursing, 2007. **16**(6): p. 1021-1028.

64. Stevens, A.B., et al., *Implementing an evidence-based caregiver intervention within an integrated healthcare system.* Translational Behavioral Medicine, 2012. **2**(2): p. 218-227.

65. Sung, H.-C., A.M. Chang, and J. Abbey, *An implementation programme to improve nursing home staff's knowledge of and adherence to an individualized music protocol.* Journal of Clinical Nursing, 2008. **17**(19): p. 2573-2579.

66. Teri, L., G. McKenzie, and D. LaFazia, *Improving dementia care in assisted living residences: addressing staff reactions to training.* Geriatric Nursing, 2009. **30**(3): p. 153-163.

67. Timmins, J., *Compliance with best practice: implementing the best available evidence in the use of physical restraint in residential aged care.* International Journal of Evidence-Based Healthcare, 2008. **6**(3): p. 345-350.

68. Tjia, J., et al., *Dissemination of evidence-based antipsychotic prescribing guidelines to nursing homes: A cluster randomized trial.* Journal of the American Geriatrics Society, 2014. **62**: p. S128.

69. van der Kooij, C., et al., *The implementation of integrated emotion-oriented care: Did it actually change the attitude, skills and time spent of trained caregivers?* Dementia: The International Journal of Social Research and Practice, 2013. **12**(5): p. 536-550.

70. van Haeften-van Dijk, A.M., et al., *Transforming nursing home-based day care for people with dementia into socially integrated community day care: process analysis of the transition of six day care centres.* International Journal of Nursing Studies, 2015. **52**(8): p. 1310-22.

71. van Haeften-van Dijk, A.M., J.C. van Weert, and R.M. Droes, *Implementing living room theatre activities for people with dementia on nursing home wards: a process evaluation study.* Aging & Mental Health, 2015. **19**(6): p. 536-47.

72. Van Mierlo, L.D., et al., *Towards personalized integrated dementia care: a qualitative study into the implementation of different models of case management.* BMC geriatrics, 2014. **14**: p. 84.

73. Van't Leven, N., et al., *Barriers to and facilitators for the use of an evidence-based occupational therapy guideline for older people with dementia and their carers.* International Journal of Geriatric Psychiatry, 2012. **27**(7): p. 742-748.

74. Vasse E, Spijker A, Meiland F, et al. *Barriers and facilitators in implementing quality indicators for psychosocial dementia care in European countries* (thesis chapter). (submitted thesis chapter). Personal communication in January 2016.

75. Vikstrom, S., et al., *A model for implementing guidelines for person-centered care in a nursing home setting.* International Psychogeriatrics, 2015. **27**(1): p. 49-59.

76. Vollmar, H.C., et al., *Knowledge transfer for the management of dementia: a cluster randomised trial of blended learning in general practice.* Implementation Science, 2010. **5**: p. 1.

77. While, C., *Supporting practice in dementia care: evaluation of an education programme for district nurses.* Journal of Mental Health Training Education and Practice, 2010. **5**(3).

78. Zwijsen, S.A., et al., *Grip on challenging behavior: process evaluation of the implementation of a care program.* Trials [Electronic Resource], 2014. **15**: p. 302.

79. Banks, P., et al., *Enriching the care of patients with dementia in acute settings? The Dementia Champions Programme in Scotland.* Dementia-International Journal of Social Research and Practice, 2014. **13**(6): p. 717-736.

80. Burgio, L.D., et al., *Teaching and maintaining behavior management skills in the nursing home.* The Gerontologist, 2002. **42**(4): p. 487-496.

81. Manthorpe, J. and K. Samsi, *Changing practice: Adapting to the Mental Capacity Act 2005.* Social Care and Neurodisability, 2013. **4**(3): p. 124-133.

82. Mellor, D., et al., *Staff Compliance With Protocols to Improve the Management of Behavioral and Psychological Symptoms of Dementia.* Journal of Gerontological Nursing, 2015. **41**(2): p. 44-52.

83. Øye, C., et al., *Evidence Molded by Contact with Staff Culture and Patient Milieu: an Analysis of the Social Process of Knowledge Utilization in Nursing Homes.* Vocations and Learning, 2015. **8**(3): p. 319-334.

84. Roberts, G., et al., *Caring for people with dementia in residential aged care: Successes with a composite person-centered care model featuring Montessori-based activities.* Geriatric Nursing, 2015. **36**(2): p. 106-110.

85. Slaughter, S.E. and C.A. Estabrooks, *Optimizing the mobility of residents with dementia: A pilot study promoting healthcare aide uptake of a simple mobility innovation in diverse nursing home settings.* BMC Geriatrics, 2013. **13**(1).

86. Verkaik, R., et al., *Introducing a nursing guideline on depression in dementia: a multiple case study on influencing factors.* International journal of nursing studies, 2011. **48**(9): p. 1129-1139.

87. Vida, S., et al., *A long-term care center interdisciplinary education program for antipsychotic use in dementia: Program update five years later.* International Psychogeriatrics, 2012. **24**(4): p. 599-605.

88. Yusoff, S., et al., *Initial evaluation of the training programme for health care professionals on the use of Malaysian clinical practice guidelines for management of dementia.* East Asian Archives of Psychiatry, 2013. **23**(3): p. 91-101.
